# Can we trust published evidence on point-of-care tests for cholesterol? A rapid review

**DOI:** 10.1136/bmjopen-2023-080726

**Published:** 2025-03-05

**Authors:** Chikomborero Cynthia Mutepfa, Timothy Patrick Hicks, Amanda Winter, Rachel Emma Dickinson, Cameron Williams, Nick Harrison, Joe Chidanyika, Julia L Newton, William Stephen Jones, Jana Suklan

**Affiliations:** 1 NIHR HealthTech Research Centre (HRC) in Diagnostic and Technology Evaluation, Newcastle Upon Tyne, UK; 2 Newcastle Upon Tyne Hospitals NHS Foundation Trust, Newcastle Upon Tyne, UK; 3 OPEN Health Group, Marlow, UK; 4 Health Innovation North East and North Cumbria (NENC), Newcastle, UK; 5 Newcastle University, Newcastle upon Tyne, UK; 6 Faculty of Science and Engineering, University of Hull, Hull, UK

**Keywords:** Lipid disorders, CARDIOLOGY, PREVENTIVE MEDICINE, Primary Prevention, Mass Screening

## Abstract

**Abstract:**

**Objectives:**

There is a need to better inform clinicians and decision-makers in primary or community care settings on selecting the appropriate point-of-care tests (POCTs) for screening purposes (as a part of the NHS Health Check Programme). Here we provide an overview of the published analytic validity and diagnostic accuracy studies on POCTs for measuring blood lipids that are available on the UK market to determine whether they meet the accuracy specifications based on the 1995 US National Cholesterol Education Program (NCEP) recommendations.

**Design:**

Rapid review of analytical validity and diagnostic accuracy studies.

**Data sources:**

On 12 May 2023, Medline and Embase were searched. Google Scholar was manually scrutinised to identify additional studies. Key article reference lists were also hand-searched.

**Eligibility criteria:**

We included analytical validity and diagnostic accuracy studies that compared POCT to laboratory testing (or another POCT) performance for measuring at least total cholesterol (TC) and high-density lipoprotein cholesterol (HDL-C).

**Data extraction and synthesis:**

Identified studies were independently reviewed by two researchers using standardised methods of screening. Where necessary, conflicts were resolved by a third reviewer. Title and abstract as well as full texts were screened using prespecified inclusion and exclusion criteria. The quality of identified studies was assessed using QUADAS-2 for diagnostic accuracy studies and a modified quality appraisal tool for studies of diagnostic reliability (QAREL) for analytical validity studies. We assessed the quality of analytical and diagnostic accuracy studies and compared the accuracy of the POCTs for TC, triglyceride (TG), HDL-C and low-density lipoprotein cholesterol (LDL-C) against NCEP standards for mean per cent bias, coefficient of variation or total error. We narratively synthesised analytical and clinical validity evidence from retrieved studies.

**Results:**

This study examined analytical and diagnostic accuracy evidence for the selected POCTs. Through the review of 22 studies, 6 POCTs were identified. All retrieved studies were analytical validity assessments, while five of them also reported diagnostic accuracy information. The majority of evidence focused on Cholestech LDX, CardioChek PA and Accutrend Plus. Evidence of between and within-study heterogeneity was found. Precision measures often showed systematic differences between the POCT and reference standards. Most devices, except for Elemark, met at least one NCEP standard for either TC, TG, HDL-C, or LDL-C.

**Conclusions:**

We found that evidence for two of the devices mostly met the requirements of the NCEP standard of evidence for bias and precision and could be recommended to general practitioners to use in the NHS Health Check programme. These were the Cholestech LDX and the Cobas b101 system.

**STRENGTHS AND LIMITATIONS OF THIS STUDY**

- We addressed concerns raised by primary care staff about the reliability of point-of-care test (POCT) results compared with laboratory testing in the NHS Health Check Programme.
- This rapid review narratively synthesises analytical and clinical validity evidence of POCTs measuring cholesterol.
- Our results provide reassurance of the performance and safety of two POCTs with similar performance characteristics as laboratory testing that could be used in clinical practice.
- This review can be used as a guide to inform healthcare professionals, managers and researchers in their decision-making when selecting the appropriate POCTs that best fit the multifaceted requirements of the setting.
- Evidence, such as clinical utility and health economics, was not considered, but may have a role to play in policymaking.

## Background

 Heart and circulatory disease, also known as cardiovascular disease (CVD), causes a quarter of all deaths in the UK and is the largest cause of premature mortality.^1 2^ The Office for Health Improvement and Disparities (formerly Public Health England) coordinates the NHS Health Check prevention programme, a screening programme providing health check-ups for adults aged 40–74.^3^ It is designed to detect early indications of various diseases, including heart disease, to identify people who would benefit from a preventive programme. It is delivered in primary care settings (general practitioners (GPs) and community pharmacies), supported by laboratory and point-of-care testing (POCT) capabilities. The early identification of people at high risk of CVD allows for appropriate management, which can lead to improved patient outcomes.^4^

The NHS Health Check guidance stipulates measurement of non-fasting blood samples for total cholesterol (TC), high-density lipoprotein cholesterol (HDL-C) and non-HDL-C (which is a subtraction of HDL-C from TC). The ratio of TC to HDL-C is then used to calculate patient risk for developing CVD using the QRISK cardiovascular risk assessment tool, together with other risk factors.^5^

POCT is testing near the patient (eg, in a patient’s home, primary care, community care, pharmacies, outpatient clinics or by the bedside in hospitals) rather than in a laboratory for rapid results that may allow for faster management/decision-making, earlier initiation and/or optimisation of treatment, no additional appointments needed, reduced incidence of complications and increased patient satisfaction.^6^,^10^ However, concerns have been raised about the reliability of POCT results compared with laboratory testing.^11 12^ Poor accuracy of POCTs for cholesterol could lead to either false reassurance or overdiagnosis and inappropriate treatment, thus potentially committing a patient to a lifetime of therapy. This might undermine the clinical effectiveness and cost-effectiveness of the NHS Health Check programme.^9^

The accuracy of POCTs can be measured in two types of studies. First, analytical validity studies which assess whether the analyte of interest is accurately and repeatably measured.^13^ Analytical validity study extraction tables usually report bias/precision and agreement/correlation. Second, diagnostic accuracy or clinical validity studies which compare results from a POCT device (index test) to those from an established laboratory method (reference standard) to determine if it can detect the disease of interest. They should be conducted in populations like those in which the test is intended to be used.^13^

Previous reviews on POCTs are now over a decade old and need updating as new POCTs become available such as the CardioChek Plus, Piccolo Xpress and Cobas b101, and others have become obsolete such as the Reflotron Plus.^14 15^ Our research question was to determine what evidence is available about the performance of the POCTs that measure cholesterol in adults compared with current reference standards. The objectives of this article were to (1) systematically identify the currently available POCTs for measuring cholesterol and (2) assess the accuracy of published evidence and compare against 1995 US National Cholesterol Education Program (NCEP) recommendations. We also aimed to assess the diagnostic accuracy of the identified POCTs.

## Methods

A rapid review was reported according to the Preferred Reporting Items for Systematic Reviews and Meta-Analyses (PRISMA) guidelines^16^ (see online supplemental appendix 1). The protocol for the study was drafted and agreed on with the funder.

### Patient and public involvement

No patients were involved in the conduct of this study.

### Literature searches and study selection

Searches were run on 12 May 2023 to systematically identify all the currently available POCTs for measuring cholesterol. The search strategy encompassed simultaneous querying through OVID, incorporating both Embase and Medline databases. The selection of searching criteria was tailored to align with the distinctive search structures of both databases (see online supplemental appendix 2). Furthermore, Google Scholar was manually scrutinised to identify any additional studies that may have been omitted in the initial search. Hand-searching of reference lists was also conducted. We used the search strategy initially used in the Buyer’s guide *Point-of-care testing for cholesterol measurement* (2009) commissioned by the NHS Purchasing and Supply Agency, Centre for Evidence-based Purchasing.^15^ We identified 37 POCTs that are used for measuring cholesterol and reviewed them against preset criteria to create a shortlist of POCTs. Brand names of the shortlisted POCTs were searched with the aim of retrieving published evidence about analytical validity and diagnostic accuracy of the tests. The identified studies were independently reviewed by two researchers (JS and CCM). Where necessary, conflicts were resolved by a third reviewer (WSJ).

### Eligibility criteria

Title and abstract as well as full texts were screened using the prespecified inclusion and exclusion criteria (eligibility criteria) for this review defined according to the Population, Intervention, Comparison, Outcome and Study Design as reported in online supplemental appendix 3.

### Data extraction

Data extracted included author, year of study, country, POCT name, target population and setting, as well as measures of precision and agreement, or diagnostic accuracy. Data were extracted by a single researcher (either JS, CCM, RED, TPH or CW), but checked for accuracy by a different member of the team who had not extracted the record. A data extraction form was developed and piloted using a smaller number of studies. A second data extraction form was developed to extract manufacturer-stated accuracy and precision from the user manuals and instructions for use.

### Quality appraisal

The quality of identified studies was assessed using the QUADAS-2^17^ for diagnostic accuracy (see online supplemental appendix 4) and modified QAREL for analytical validity studies by a pair of reviewers responsible for data extraction and validation (JS, CCM, RED, TPH, CW).^18^ We modified the QAREL tool with additional questions (see online supplemental appendix 5). Discrepancies were resolved through discussion, where necessary conflicts were resolved by a third reviewer (WSJ or TPH).

### Accuracy assessment

Results on precision, concordance (for analytical validity studies), and sensitivity, specificity, area under the receiver operating characteristic curve (AUROC) (used to evaluate model’s performance) and predictive values (for clinical validity studies) were extracted. Extracted data were described and summarised, similarities and differences between studies were reported, and relationships were explored. For the analysis of precision, the mean (percentage) bias, coefficient of variation (CV) and total error (TE) of TC, TG, HDL-C and LDL-C, if provided, were compared against the NCEP Laboratory Standardization Panel guidelines^19^ (see online supplemental appendix 6). Meta-analysis was not appropriate due to heterogeneity of evidence identified for each of the POCTs.

## Results

### Study selection

**Figure 1:**
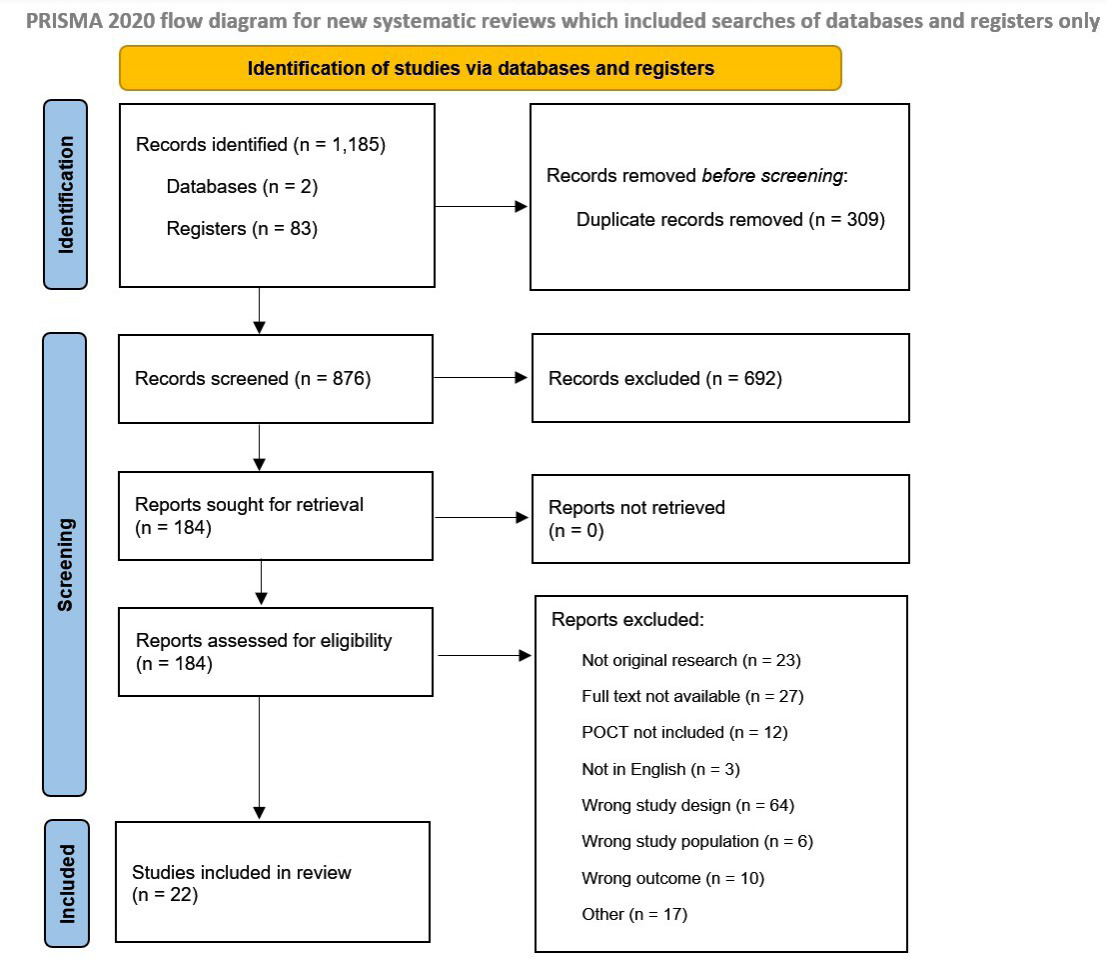
Preferred Reporting Items for Systematic Reviews and Meta-Analyses 2020 flow diagram template for systematic reviews. POCT, point-of-care testing. Source: Page *et al*.^16^ This work is licensed under CC BY 4.0. To view a copy of this license, visit https://creativecommons.org/licenses/by/4.0/.

A total of 22 studies were included in the final analysis after identifying 1185 articles from the search strategy and screening 876 de-duplicated articles based on eligibility criteria. The PRISMA flow diagram of the included and excluded studies is shown in figure 1. A PRISMA extension checklist for diagnostic test accuracy (DTA) studies items is presented in online supplemental appendix 1.

### Characteristics of the included studies

The evidence for the six POCTs is summarised in table 1. Features and specifications of the selected cholesterol POCT devices are presented in online supplemental appendix 8. The evidence was unevenly distributed across POCTs, with most of the included studies focusing on three POCTs (Cholestech LDX (n=7), CardioChek PA (n=8) and Accutrend Plus (n=6)). Cobas b101, Elemark and Afinion AS100 each have three or fewer published studies reporting on their performance. Studies were conducted in Australia, Belgium, Brazil, Italy, Kenya, South Africa, South Korea, Spain, Switzerland, the Netherlands, the UK and the USA. The study participants were mostly healthy volunteers recruited from secondary care. Other participants were recruited from clinics within primary or tertiary care. A few studies (n=6) did not report the fasting status of participants.

**Table 1:** Study characteristics

Index testPOCT name, manufacturer, country	Reference	Setting/patient population	Reference standard
Accutrend Plus, Roche Diagnostics, UK	Barrett *et al*, Australia^35^	Laboratory, secondary care; 40 non-fasting women in late pregnancy.	National Association of Testing Authorities, Australia accredited to ISO 15189 standard clinical, Beckman DXC800, venous blood and capillary blood
	Coquiero *et al*, Brazil^20^	Secondary care; 53 non-fasting adults.	Private clinical laboratory Diagnóstica Laboratório de Análises Clínicas Ltda (Labmax 240), venous blood
	Kurstjens *et al*, Netherlands^43^	Secondary care; 61 adults. Fasting state not reported.	Dutch Foundation for Quality Assessment in Medical Laboratories (SKML), chemistry analyser (Advia Chemistry XPT system, Siemens, Germany) at the JBZ clinical chemistry laboratory, JBZ (Siemens, Advia Chemistry XPT system), routine venous blood sample
	Scafoglieri *et al*, Belgium^21^	Laboratory, secondary care; 60 healthy, fasting volunteers.	Vitros 5.1 FS chemistry system, venous blood
	Mendez-Gonzalez *et al*, Spain^22^	Laboratory, secondary care, 109 healthy volunteers. Fasting state not reported.	Accutrend GCT, capillary (71) CardioChek PA, venous (109)Hitachi 912 instrument as reference, venous blood
	Maciel *et al*, Brazil^23^	Community; 30 fasting, adult healthy volunteers.	Laboratory tests, venous blood
Cobas b101 System, Roche Diagnostics, Switzerland	Barroso *et al*, Spain^40^	Secondary care; 937 fasting, adults aged 35–74, patients at risk of cardiovascular disease (≥5%).	Pentra autoanalyzer, venous blood
	ICCnet CHSA, Australia^36^	Primary care; 140 fasting adults.	Local Laboratory, venous blood
	Ordonez-Llanos/Roche *et al*, Spain and Switzerland^37^	Laboratory, 160 adults.Fasting state not reported.	Cobas 6000+c 501 module using Li-heparin whole blood
Afinion AS100, Abbott, USA	Abbai *et al*, South Africa^24^	Research clinic; 435 non-fasting, adults ≥50 years age.	ABX Pentra 400, venous blood
Cholestech LDX, Abbott, USA	Bastianelli *et al*, USA^25^	Laboratory; 30 fasting adults.	Venepuncture standard lipid panel, measured using Integra and Cobas analysers, venous blood
	Donato *et al*, USA^26^	Laboratory; 57 fasting and non-fasting, healthy volunteers.	Cobas c501, venous serum
	Jain *et al*, UK^41^	Community screening; 406 fasting and non-fasting South Asian adults aged 30–74 years.	Roche Modular P analyser, venous blood
	O'Donovan *et al*, Ireland^27^	Laboratory; 80 healthy volunteers. Fasting state not reported.	Reflotron Plus analysis, capillary blood
	Parikh *et al*, USA^28^	Laboratory; 250 healthy fasting family members or cohabitants of patients hospitalised with CVD, between 20 and 79 years of age.	Columbia University General Clinical Research Center core laboratory by using standard methods for serum lipids, venous blood
	Whitehead *et al*, UK^29^	Laboratory; 167 non-fasting adults in the NHS Health Checks clinics.	Roche Modular P analyser, CardioChek PA, venous blood
	Whitehead *et al*, UK^30^	Laboratory; 162 adults at community health check clinics. Fasting state not reported.	Roche Modular P analyser, CardioChek PA, venous blood.
CardioChek PA Analyzer, Polymer Technology Systems, USA	Mendez-Gonzalez *et al*, Spain^22^	Laboratory, secondary care; 109 adults. Fasting state not reported.	Accutrend GCT, capillary bloodAccutrend Plus, capillary blood (71)Hitachi 912 instrument as reference, venous blood
	Bolodeoku *et al*, UK^39^	Laboratory, secondary care; one fasting, healthy adult.	Elemark, capillary blood
	Bolodeoku *et al*, UK^38^	Laboratory, secondary care; three non-fasting, healthy adults.	Elemark, venous blood
	Donato *et al*, USA^26^	Laboratory; 57 fasting and non-fasting healthy volunteers.	Cholestech LDX, capillary bloodCholestech, residual serumCobas c501, venous serum
	dos Santos Ferreira *et al*, Brazil^42^	Laboratory; 516 fasting, healthy volunteers.	Cobas 6000, venous blood
	Park *et al*, Kenya^31^	Community; 246 fasting and non-fasting (5%) adults screened for non-communicable diseases.	Cobas INTEGRA 400 plus Biochemistry Analyzers, venous blood
	Whitehead *et al*, UK^29^	Laboratory, primary care; 167 non-fasting individuals screened for diabetes and CVD.	Modular P analyser, venous bloodCholestech LDX, capillary blood
	Whitehead *et al*, UK^30^	Laboratory, community care; 162 adults at the NHS Health Checks clinics. Fasting state not reported.	Cholestech LDX, capillary blood; Modular P analyser, venous blood
Elemark, BBB Tech, South Korea	Bolodeoku *et al*, UK^39^	Laboratory, secondary care; one healthy fasting individual.	CardioChek PA, capillary blood 3in1, capillary blood
	Bolodeoku *et al*, UK^38^	Laboratory, secondary care; three healthy non-fasting individuals.	CardioChek PA, venous blood
	Yun *et al*, South Korea^32^	Laboratory, community; 116 healthy adults. Fasting state not reported.	AU5800 Analyzer, venous serum

CVDcardiovascular diseasePOCTpoint-of-care testing

### Quality assessment

#### Modified QAREL for analytical validity studies

The quality of analytical validity studies was heterogeneous, with all studies meeting some requirements, but not all. A modified quality assessment tool for the analytical validity studies is presented in online supplemental appendix 5. All studies were conducted prospectively, with 14 being cross-sectional,^20^,^34^ 6 were cohort studies,^2122 35^,^38^ 1 case study^39^ and 1 randomised controlled study,^40^ thus meeting the criteria for a prospective study design.

All other criteria had some risk of bias present; mostly associated with the lack of adequate repetition of measures or the use of dissimilar sample types for analysis between the index test and reference standard which could introduce detection and performance biases. The potential for sponsor bias was evident, as full, or partial funding for the studies was either provided by the manufacturer or not disclosed.^21 22 25 31 32 36 37 39 41 42^ In addition, a few studies stated that they had not received funding but had equipment donated by the manufacturer.^22 28^ Domains relating to the reference standard including use of an appropriate measurement range or sample type; or a quality control for the reference standard, or if it met requirements for established guidelines (such as the NCEP/Cholesterol Reference Method Laboratory Network/National Health Laboratory Service or similar) were mostly unreported in the papers, introducing reporting biases. Almost all studies did not provide clear reporting on questions related to blinding of the raters, and only Scafoglieri and others’^21^ evaluation of between-day results from Accutrend blinded raters to their own prior findings. All authors evaluated their tests in a sample of subjects who were representative of those to whom the authors intended the results to be applied. Finally, most (n=15) tests were conducted by healthcare professionals, while it was unclear who conducted the tests in the other studies.

Whitehead studies^29 30^ had the lowest risk of bias domains, whereas the Yun^32^ study had five domains with a high risk of bias: the highest. The only limitation in Whitehead’s evaluation of the diagnostic accuracy of CardioChek PA and Cholestech LDX in CVD risk assessment was insufficient measurement replication.^36 40^ Contrastingly, in its assessment of the Elemark smartphone device from the general adult population, Yun *et al*.’s quality control was not performed for either the index or the reference standard test; adequate measurement ranges were not used, samples were not analysed in triplicate and funding for the study was provided by the manufacturer.^32^

#### QUADAS-2 for diagnostic accuracy studies

The quality assessment tool for the diagnostic accuracy studies is presented in online supplemental appendix 4. Overall, convenience sampling techniques were used in the recruitment of patients 18 years and above in different settings and the targeted population were applicable to our review. Thus, there was little or no concern over the conduct of patient selection and the index test in the diagnostic accuracy studies. However, there were some concerns about how the reference tests were conducted and the flow and timing of the events, which could have introduced partial verification and incorporation biases. In most cases, it was unclear how the reference standard testing was carried out and if investigators had prior knowledge of the index test results in Abbai and colleagues’^24^ evaluation of the Afinion AS100, and Coquiero *et al*, Kurstjens *et al*, and Maciel *et al*’s evaluation of Accutrend Plus.^20 23 43^ In the Parikh and colleagues’ study,^28^ reference standard review bias^17^ was introduced by sending the Cholestech LDX analyser results to the participants’ primary care physician.

The studies by Coquiero and Maciel^20 23^ had the least risk of bias while Abbai’s had the highest risk of bias.^24^ Abbai and colleagues’ paper^24^ on the Afinion AS100 analyser had the potential for selection, disease progression and reference standard review biases because it was unclear whether the recruitment was conducted consecutively and how long the time difference was between the conduct of the index test and reference standard. Additionally, not all 435 patients recruited were included in the analysis.^17^ Kurstjen’s study^43^ also had a high risk of bias introduced by the lack of clarity on the interpretation of the reference standard and the exclusion of some participants from analysis. While they provided a reason for the exclusion of participants in the analysis of the Mission 3-in-1 POCT, an explanation for Veroval’s analysis of 59/61 participants was omitted.

There were generally no concerns with the applicability of the test in relation to the review aims.

### Analytical and clinical validity of the POCTs

Almost all studies assessed POCT performance for TC measurements. Only one paper by Donato *et al*^26^ reported non-HDL-C metrics. The outcome measures for accuracy were varied, with different aspects of analytical validity, and different measures of precision and agreement being presented as shown in table 2. The outcome measures for clinical validity studies are shown in table 3.

**Table 2:** Outcome measures for analytical precision of POCTs

POCT	Reference	Mean bias (mmol/L), [Limits of agreement], (95% CI), mean per cent bias, *±SD*	Coefficient of variation (%)	Total error (%)
Accutrend Plus, Roche Diagnostics	Barrett *et al*^35^	TG: −0.01 [−0.93, –0.91], *−0.5 (−28.5* to *27.5)*		
	Scafoglieri *et al*^21^	TC: 0.26 [−0.95, 1.47], (0.10 to 0.42)TG: −0.16 [−1.29, 0.98], (−0.32 to 0.01)		
	Mendez-Gonzalez *et al*^22^	TC: *−8*TG: *8.8*		TC: 8.5TG: not reported
	Maciel *et al*^23^	TC: 0.48 [-1.04, 2.01]TG: 0.29 [-2.45, 3.05]		
Cobas b101 System, Roche Diagnostics, Switzerland	ICCnet CHSA^36^	TC: *−5.71*TG: *3.50*HDL-C: *−1.43*		
	Ordonez-Llanos/Roche *et al*^37^	**Capillary whole blood (WB**)TC: *−0.54*TG: *3.88*HDL-C: *2.06***EDTA WB**TC: −*1.94*TG: *0.13*HDL-C: *−0.15*	**Capillary WB**TC: 1.66TG: 1.39HDL-C: 2.3**EDTA WB**TC: 1.55TG: 1.38HDL-C: 2.08	**Capillary WB**TC: 2.71TG: 6.60HDL-C: 6.57**EDTA WB** TC: 1.10TG: 2.83HDL-C: 3.93
Afinion AS100, Abbott, USA	Abbai *et al*^24^	TC: 0.569 [−0.169, –1.307], *±0.377*LDL-C: 0.528 [−0.227, –1.283], *±0.385*HDL-C: 0.089 [−0.097, –0.275] *±0.095*TG: –0.124 [−0.373, –0.125], *±0.127*		
Cholestech LDX, Abbott, USA	Bastianelli *et al*^25^	**vs Integra analyser**TC: *0.5*HDL-C: *4.5*TG: *3.3***vs Cobas analyser**TC: *4.6*HDL-C: *2.6*TG: *1.6*		
	Donato *et al*^26^	TC: *−7.0±6.3*TG: *5.2±38.0*HDL-C: *−12.5±9.8*Non-HDL-C: *−4.4±7.6*LDL-C: *−5.5±10.4*		
	O’Donovan *et al*^27^	TC: 0.24 [0.15, 0.33]HDL-C: 0.24 [0.16, 0.33]TG: 0.10 [0.01, 0.19]LDL-C: 0.05 [0.11, 0.23]		
	Whitehead *et al*^29^	TC: *−0.14±0.13*HDL-C: *0.7±0.7*TG: *−0.8±0.6*		
	Whitehead *et al*^30^			TC: 8.4HDL-C: 15.4
CardioChek PA Analyzer, Polymer Technology Systems, USA	Mendez-Gonzalez *et al*^22^			TC: 25.1TG: 14HDL-C: 18.4
	Bolodeoku *et al*^39^	TC: 4.2*±0.4*TG: 0.8*±0.2*HDL-C: 1.5*±0.1*LDL-C: 2.4*±0.3*	TC: 9.4TG: 23HDL-C: 7LDL-C: 14	
	Bolodeoku *et al*^38^		TC: 8TG: 22.4HDL-C: 8LDL-C: 9.4	
	Donato *et al*^26^	TC: *−7.5±11.1*TG: *−2.6±36.0*HDL-C: *−3.9±14.5*non-HDL-C: *−9.5±15.9*LDL-C: *−10.0±21.6*		
	dos Santos Ferreira *et al*^42^	**Capillary**TC: *−3.3*HDL-C: *7.5*TG: *12.7***Venous**TC: −7HDL-C: *9*TG: *4.5*		
	Park *et al*^31^	TC: *−15.9 *(−19.8, –12.1)TG: *0.03 *(−8.6, 8.6)HDL-C: *−8.2 *(−12.9, –3.6)LDL-C: *−25.9 *(−29.7, –22.1)	TC: −0.22TG: −0.18HDL-C: 0.29LDL-C: −0.27	
	Whitehead *et al*^29^	TC: *−12.7±18.8*HDL-C: *1.7±15.8*	TC: 15.1 (13–19.3)HDL-C: 18.9 (17.5–20.3)	
	Whitehead *et al*^30^			TC: 25HDL-C: 25.1
Elemark, BBB Tech, South Korea	Bolodeoku *et al*^39^		TC: 5TG: 30HDL-C: 13LDL-C :13	
	Bolodeoku *et al*^38^		TC: 4TG: 30.3HDL-C: 14LDL-C: 15.3	

Values in column 3 are displayed as: Mean bias, [Limits of agreement], (95% CI), *mean per cent bias*, *±SD*

HDL-C, high-density lipoprotein cholesterol; LDL-C, low-density lipoprotein cholesterol; POCTpoint-of-care testingTC, total cholesterol; TGtriglycerideWB, whole blood

**Table 3:** Outcome measures for diagnostic accuracy performance of POCTs

POCT	Reference	Type of lipid measured	Diagnostic accuracy (%)
Accutrend Plus, Roche Diagnostics	Coquiero *et al*^20^	TC ☒TG ☒HDL-C ☐LDL-C ☐Non-HDL-C ☐	*Diagnostic threshold (mmol/L): TC=5.17; TG=1.69***TC**Sensitivity: 84.4Specificity: 95.2**TG**Sensitivity: 90.5Specificity: 96.9
	Kurstjens *et al*^43^	TC ☒TG ☐HDL-C ☐LDL-C ☐Non-HDL-C ☐	*Diagnostic threshold (mmol/L): TC=5.17*Sensitivity: 92Specificity: 89Positive predictive value: 85Negative predictive value: 94
	Maciel *et al*^23^	TC ☒TG ☒HDL-C ☐LDL-C ☐Non-HDL-C ☐	*Diagnostic threshold (mmol/L): TC=5.17; TG=1.69***TC**Sensitivity: 100Specificity: 69.2**TG**Sensitivity: 100Specificity: 80
Cobas b101 System, Roche Diagnostics, Switzerland	Barroso *et al*^40^	TC ☒TG ☒HDL-C ☒LDL-C ☒Non-HDL-C ☐	Threshold Cardiovascular risk>5%*Male*:Sensitivity, % (95% CI): 0.74 (0.63 to 0.82)Specificity, % (95% CI): 0.97 (0.95 to 0.99)Accuracy (95% CI): 0.93 (0.90 to 0.95)*Female*:Sensitivity, % (95% CI): 0.50 (0.30 to 0.70)Specificity, % (95% CI): 0.99 (0.98 to 1.00)Accuracy (95% CI): 0.97 (0.95 to 0.98)
Cholestech LDX, Abbott, USA	Parikh *et al*^28^	TC ☒TG ☒HDL-C ☒LDL-C ☒Non-HDL-C ☐	*Diagnostic threshold (mmol/L): TC≥5.17; LDL-C^1^≥2.59; LDL-C^2^≥3.36, HDL-C<1.03; TG≥1.69***TC**Sensitivity: 79Specificity: 95**LDL-C**^**1**^Sensitivity: 93Specificity: 82**LDL-C**^**2**^Sensitivity: 76Specificity: 92**HDL-C**Sensitivity: 93Specificity: 78**TG**Sensitivity: 88Specificity: 93

HDL-Chigh-density lipoprotein cholesterolLDL-Clow-density lipoprotein cholesterolPOCTpoint-of-care testingTCtotal cholesterolTGtriglyceride

Overall, distinctive differences in the evidence for quality and accuracy of POCT for cholesterol measurement have been found in our review. This ambiguity creates challenges in effectively comparing them head-to-head. We found some discrepancies in the reporting of results for studies assessing multiple POCTs whereby some outcome measures were not reported uniformly for all POCTs under investigation. For example, in Mendez- Gonzales,^22^ although they provided a total inaccuracy/bias measurement for all POCTs, some measures were either reported for the CardioChek PA and not for Accutrend Plus, at different cut-off points, and for different samples (either patient blood or control material) in the TG measurements.

The mean per cent bias was reported for TC and TG for four POCTs: Accutrend Plus, CardioChek PA, Cholestech LDX and Cobas b101. The mean per cent bias for HDL-C was reported for three POCTs: CardioChek PA, Cholestech LDX and Cobas b101. Only one POCT, CardioChek PA, had papers that reported the mean per cent bias for LDL-C, and these were both beyond the ±4% limits for this endpoint. No mean per cent bias was reported for Afinion and Elemark; however, the mean bias was provided for the Afinion.

CVs were reported for four POCTs—CardioChek PA, Cholestech LDX, Cobas b101 and Elemark—from eight studies. Cobas b101 met the NCEP standards for TC, TG and HDL-C, although only one study provided CVs for Cobas b101. Cholestech LDX only met NCEP standards for TC and TG, while the rest of the POCTs did not meet any standards. There were no CVs reported for LDL-C.

Very few studies reported on the TEs for each POCT, and not all cholesterol endpoints were included. A total of five papers reported TE on Accutrend, CardioChek PA, Cholestech LDX and Cobas b101. Accutrend and Cobas b101 met the NCEP standard for TC. CardioChek PA, Cholestech and Cobas b101 met the NCEP standard for TG, while Cobas b101 also met the standard for HDL-C. Only one paper reported on the TE for LDL-C which was for the Cholestech POCT, and this was above the NCEP standard.

On average, studies for the POCTs Cholestech LDX and Cobas b101 reported mean per cent bias, CV and TE ranges that all met the NCEP standard for TC, TG and HDL-C. On the other hand, papers on Elemark reported CVs that did not meet any of the standards.

Diagnostic accuracy for the different cholesterol endpoints was reported for some of the POCTs inconsistently, and the diagnostic thresholds were similar in most studies for the different analytes except for TC. A cut-off point of 4.5 mmol/L was used in the Abbai study, according to South African guidelines, compared with the accepted 5.2 mmol/L used in other studies. The sensitivity and specificity for TC were reported for Accutrend Plus, Afinion AS100, Cholestech LDX and Cobas b101. The sensitivity and specificity to HDL-C were measured for Afinion AS100 and Cholestech LDX, and excellent sensitivity and specificities to TG were reported for the Accutrend Plus, Afinion As100 and Cholestech LDX.

Extracted data on agreement (for analytical validity studies) are reported in online supplemental appendix 7. Summaries of the analytical performance followed by the diagnostic accuracy performance for each POCT are provided below in a narrative format.

#### Accutrend Plus

All five Accutrend Plus papers assessed analytical validity and three were also clinical validity studies from a total of 353 participants. As shown in table 2, the mean bias for TC and TG was reported in the three studies. Two of these reported the mean per cent bias of −0.5% and 8.8% for TG. The mean per cent bias for TC of −8% was reported in one paper,^22^ while the other two reported in mmol/L. Mendez-Gonzalez and colleagues^22^ was the only study to report TE percentage for TC, and this was well above the NCEP recommendations at 8.5%. In the analyses for agreement, good to excellent correlations were mostly found for TC and TG between the Accutrend Plus and reference standard methods.

Diagnostic accuracies were reported in two of the studies and were measured at similar cut-off points. The sensitivity and specificity for both TG and TC were high.

#### Afinion AS100

One paper, evaluating 435 people by Abbai and colleagues reported on both the analytical and diagnostic performance of the Afinion AS100^24^ for measuring TC, TG, HDL-C and LDL-C analytes. The mean biases were narrowly spread as shown by the limits of agreement in table 2. Lin’s concordance coefficient was used to assess overall agreement and correlation and showed that TC, TG, HDL-C and LDL-C had good to excellent correlation with the reference standard. Deming regression was used in Jain’s paper to assess agreement; however, no measure for correlation or significance was provided. Passing Bablok regression was reported and showed excellent agreement and correlation between the Afinion and the laboratory for TC, TG, HDL-C and LDL-C as r values were >0.95.

In the analysis of the diagnostic accuracy of Afinion, the sensitivity values for all cholesterol endpoints were above 90% for men and women. The specificity differed, where for TC and LDL-C these were approximately 63% for both sexes, while the specificity of HDL-C was higher in women at 80.9%, compared with 63.2% for men. The specificity for TG was 100% for both men and women.

#### CardioChek PA

Eight analytical validity studies were analysed from a total of 1261 participants. The mean bias and CVs were reported in most studies, and these were widely dispersed for all clinical endpoints. The mean bias was reported in mmol/L in one study and as percentages in five studies. The mean per cent bias for TC, TG and HDL-C ranged between −15.9% to 6.5%, −3.3% to 12.7% and −8.2% to 10.3%, respectively. The mean per cent bias for LDL-C was reported in two studies;^26 31^ both exceeded NCEP recommendations at −10.0% and 25.9%. The CVs ranged between −0.22% to 15.1%, −0.18% to 23%, 0.29 to 18.9% and −0.27 to 14% for TC, TG, HDL-C and LDL-C, respectively.

The TE was reported in two studies, with results well above the recommended NCEP guidelines for TC and HDL-C in two of the studies.^22 30^ A TE of 14% for TG, just within NCEP limit, was also reported in one study.^22^

Agreement between the POCT and reference standard was explored in two studies using either linear regression or Pearson’s correlation coefficient. Both papers reported strong positive correlations between POCT and reference standard.

#### Cholestech LDX

1152 people in differing settings were recruited in the evaluations of Cholestech LDX in the seven evaluations.

The mean per cent bias for TC and HDL-C was most reported and widely differed across the studies by Bastianelli, Donato and Whitehead.^25 26 29^ The mean bias for TC ranged from −7.4% to 0.5%; while it ranged between −12.5% to 4.5% for HDL-C and was between −0.8% to 5.2% for TG. The TEs were reported by Whitehead and colleagues^30^ and were within the limits for TC analyte and above the NCEP recommendation for HDL-C. Based on these ranges, the measurement of TG was the only clinical endpoint to meet NCEP recommendations for this device. The other paper reporting on analytical validity by Jain and colleagues^41^only reported Deming regression to assess agreement; however, no measure for correlation or significance was provided.

Only one paper reported diagnostic accuracy for this POCT, and these were specific to CVD risk classification.^28^ It showed good sensitivities and specificities for TC, TG, HDL-C and LDL-C, although at higher concentrations the sensitivity of LDL-C decreased, while its specificity increased.

#### Cobas b101

Three studies assessed the performance of the Cobas b101 from a total of 1237 participants.^36 37 40^ Two of them reported the mean bias for TC, TG and HDL-C, and these differed for TC, but were within the NCEP standards for TG and HDL-C.^36 37^ The other study only reported concordance.^40^ Good to excellent correlations of more than 0.8 were found between the POCT and reference laboratory standards for all clinical endpoints. Barroso *et al* presented diagnostic accuracy results based on a threshold for cardiovascular risk, so are incomparable to other studies.

#### Elemark

Three studies reported on the performance of Elemark involving 120 participants.^32 38 39^ The two studies by Bolodeoku^38 39^ reported the CV involving less than 5 participants. The CVs for TC, TG, HDL-C and LDL-C averaged 4.5%, 30.15%, 13.5% and 14.15%, respectively, and did not fall within NCEP standards. On the other hand, Yun^32^ reported excellent correlation between POCT and reference standard using linear regression for TC, TG and HDL-C, respectively.

## Discussion and conclusions

### Key findings

We have systematically identified published, peer-reviewed analytical and clinical evidence on POCTs for measuring cholesterol. We identified 22 analytical validity studies and 5 clinical validity studies associated with 6 POCTs, namely the Afinion AS100, Accutrend Plus, CardioChek PA, Cholestech LDX, Cobas b101 and Elemark. We compared the mean per cent bias, CV and TE results where available against the NCEP guidelines to show concordance of classification between the POCT and laboratory. In addition, the sensitivity and specificity results of POCTs were compared. There was wide heterogeneity in the reporting of and the results for the other POCTs which lessened our consideration of their analytical and diagnostic performance. In general, from the studies presented, the results for TC measurements varied greatly, but TG and HDL-C measures were more consistent among POCTs, especially for the Cobas b101 and Cholestech LDX.

This builds on previously published work. Plüddemann and colleagues first reviewed Cholestech LDX and CardioChek PA for GP management of CVD,^44^ then Haggerty and Tran focused on POCTs for cholesterol measurement in pharmacies.^14^

### Strengths and weaknesses of the study

Our exclusion criteria did not specify which lipids the POCT should measure, which resulted in our inclusion of Accutrend Plus. As Accutrend Plus only measures TC and TG, it would not be very useful for a GP to use as part of the NHS Health Check. At a minimum, an ideal POCT should measure TC and HDL-C as the non-HDL-C can then be calculated by subtracting HDL-C from TC, and they can be used in the calculation of a QRISK-3 score.^45^

Furthermore, we reviewed the evidence for Piccolo Xpress^46 47^ which suggested that it would be most useful for NHS Health checks as its results for TC, TG and HDL-C that were consistently within the NCEP standards. The mean biases and CVs for TC, HDL-C and TG were marginally within the NCEP levels. The TEs for TC, TG and HDL-C were also reported in one study, and all were within the NCEP limits. However, the evidence was gathered on the results of two analytical validity studies, with a collective sample size of 112 patients with diabetes. As per exclusion criteria, NHS Health checks are intended for adults without preexisting CVD, making this cohort deviate from the intended clinical use population.

Due to the paucity of studies for some POCTs and heterogeneity of reporting, the evidence has been presented in a narrative format and has not been fully synthesised through a meta-analysis of each POCT. To overcome this issue, the original authors for each paper could be contacted to obtain more data; however, budget and time constraints precluded this.

### Strengths and weaknesses of the available evidence

The evidence is unevenly distributed across POCTs, with most of the included evidence focused on three POCTs (Cholestech LDX, CardioChek PA, Accutrend Plus), leaving the other POCTs with no more than three published studies reporting on their performance. More evidence was available for POCTs that have been around longer, like findings in other reviews.^48^ However, a larger evidence base may not establish how accurately a device performs as illustrated by Cholestech LDX, which had the largest number of studies and participants altogether but wider ranges in bias and imprecision reported.

There was wide heterogeneity in the types of samples and reporting of results that could lessen generalisability. This deficiency in the quality of studies in these domains was similarly found in a previous review of POCTs in community pharmacies.^49^ None of the studies followed any reporting guidelines or checklist for reporting diagnostic accuracy studies, although the Standards for Reporting Diagnostic accuracy studies (STARD) 2015 guidelines have been available since 2003.^50^ On the other hand, a checklist for analytical validity studies (The Laboratory Evaluation and Analytical Performance Characteristics (LEAP)) has only recently been published in 2024.^51^

A low volume of published material available led us to include studies from a broad geographical range, encompassing both low- and middle-income countries as well as high-income countries. While this approach allowed for a more comprehensive review of the existing evidence, it also introduced heterogeneity in healthcare systems and patient populations. These differences could potentially influence the generalisability of findings to our target population and complicate direct comparisons across settings. Furthermore, the inclusion of studies from diverse regions reflects the variations in reporting quality and methodologies, which affects the robustness of our conclusions.

### Implications for policy and practice

Cholestech LDX and Cobas b101 have evidence that suggest that they are the most accurate and precise for HDL-C and TG analytes.

### Unanswered questions and future research

Other evidence related to health economics and usability is also worth consideration. Understanding the economic impact and usability of these devices can further inform their potential effectiveness and real-world applicability.

To enhance the consistency and transparency of reporting in analytical validity and clinical validity studies, it is also necessary to follow standardised reporting guidelines. Reporting guidelines such as LEAP and STARD have been developed to encourage this. It would be interesting to determine their usage in studies a decade or so after publication in future reviews.

## supplementary material

10.1136/bmjopen-2023-080726 online supplemental file 1

## Data Availability

All data relevant to the study are included in the article or uploaded as supplementary information.
