# Supplementary material for: Can we trust published evidence on point-of-care tests for cholesterol? A rapid review
Source: BMJ Open. 2025 Mar 5;15(3):e080726. doi: 10.1136/bmjopen-2023-080726 (PMC11883607; doi:10.1136/bmjopen-2023-080726)
Supplement: online supplemental file 1 [file bmjopen-15-3-s001.docx]

# Appendix 1: PRISMA - DTA Checklist

| Section/topic | # | PRISMA-DTA Checklist Item | Reported on page # |
| --- | --- | --- | --- |
| TITLE / ABSTRACT | | |  |
| Title | 1 | Identify the report as a systematic review (+/- meta-analysis) of diagnostic test accuracy (DTA) studies. | 1 |
| Abstract | 2 | Abstract: See PRISMA-DTA for abstracts. | 2-3 |
| INTRODUCTION | | |  |
| Rationale | 3 | Describe the rationale for the review in the context of what is already known. | 5 |
| Clinical role of index test | D1 | State the scientific and clinical background, including the intended use and clinical role of the index test, and if applicable, the rationale for minimally acceptable test accuracy (or minimum difference in accuracy for comparative design). | 5 |
| Objectives | 4 | Provide an explicit statement of question(s) being addressed in terms of participants, index test(s), and target condition(s). | 5 |
| METHODS | | |  |
| Protocol and registration | 5 | Indicate if a review protocol exists, if and where it can be accessed (e.g., Web address), and, if available, provide registration information including registration number. | 5 |
| Eligibility criteria | 6 | Specify study characteristics (participants, setting, index test(s), reference standard(s), target condition(s), and study design) and report characteristics (e.g., years considered, language, publication status) used as criteria for eligibility, giving rationale. | 6, Appendix 3 |
| Information sources | 7 | Describe all information sources (e.g., databases with dates of coverage, contact with study authors to identify additional studies) in the search and date last searched. | 6 |
| Search | 8 | Present full search strategies for all electronic databases and other sources searched, including any limits used, such that they could be repeated. | Appendix 2 |
| Study selection | 9 | State the process for selecting studies (i.e., screening, eligibility, included in systematic review, and, if applicable, included in the meta-analysis). | 6 |
| Data collection process | 10 | Describe method of data extraction from reports (e.g., piloted forms, independently, in duplicate) and any processes for obtaining and confirming data from investigators. | 6 |
| Definitions for data extraction | 11 | Provide definitions used in data extraction and classifications of target condition(s), index test(s), reference standard(s) and other characteristics (e.g. study design, clinical setting). | Appendix 3 |
| Risk of bias and applicability | 12 | Describe methods used for assessing risk of bias in individual studies and concerns regarding the applicability to the review question. | 6 |
| Diagnostic accuracy measures | 13 | State the principal diagnostic accuracy measure(s) reported (e.g. sensitivity, specificity) and state the unit of assessment (e.g. per-patient, per-lesion). | 6 |
| Synthesis of results | 14 | Describe methods of handling data, combining results of studies and describing variability between studies. This could include, but is not limited to: a) handling of multiple definitions of target condition. b) handling of multiple thresholds of test positivity, c) handling multiple index test readers, d) handling of indeterminate test results, e) grouping and comparing tests, f) handling of different reference standards | 6-7 |

| Section/topic | # | PRISMA-DTA Checklist Item | Reported on page # |
| --- | --- | --- | --- |
| Meta-analysis | D2 | Report the statistical methods used for meta-analyses, if performed. | NA |
| Additional analyses | 16 | Describe methods of additional analyses (e.g., sensitivity or subgroup analyses, meta-regression), if done, indicating which were pre-specified. | NA |
| RESULTS | | |  |
| Study selection | 17 | Provide numbers of studies screened, assessed for eligibility, included in the review (and included in meta-analysis, if applicable) with reasons for exclusions at each stage, ideally with a flow diagram. | 7 |
| Study characteristics | 18 | For each included study provide citations and present key characteristics including: a) participant characteristics (presentation, prior testing), b) clinical setting, c) study design, d) target condition definition, e) index test, f) reference standard, g) sample size, h) funding sources | 7-9 |
| Risk of bias and applicability | 19 | Present evaluation of risk of bias and concerns regarding applicability for each study. | 9-10 |
| Results of individual studies | 20 | For each analysis in each study (e.g. unique combination of index test, reference standard, and positivity threshold) report 2x2 data (TP, FP, FN, TN) with estimates of diagnostic accuracy and confidence intervals, ideally with a forest or receiver operator characteristic (ROC) plot. | NA |
| Synthesis of results | 21 | Describe test accuracy, including variability; if meta-analysis was done, include results and confidence intervals. | 10-14 |
| Additional analysis | 23 | Give results of additional analyses, if done (e.g., sensitivity or subgroup analyses, meta-regression; analysis of index test: failure rates, proportion of inconclusive results, adverse events). | NA |
| DISCUSSION | | |  |
| Summary of evidence | 24 | Summarize the main findings including the strength of evidence. | 16-17 |
| Limitations | 25 | Discuss limitations from included studies (e.g. risk of bias and concerns regarding applicability) and from the review process (e.g. incomplete retrieval of identified research). | 16-17 |
| Conclusions | 26 | Provide a general interpretation of the results in the context of other evidence. Discuss implications for future research and clinical practice (e.g. the intended use and clinical role of the index test). | 16-17 |
| FUNDING | | |  |
| Funding | 27 | For the systematic review, describe the sources of funding and other support and the role of the funders. | 3 |

Adapted From: McInnes MDF, Moher D, Thombs BD, McGrath TA, Bossuyt PM, The PRISMA-DTA Group (2018). Preferred Reporting Items for a Systematic Review and Meta-analysis of Diagnostic Test Accuracy Studies: The PRISMA-DTA Statement. JAMA. 2018 Jan 23;319(4):388-396. doi: 10.1001/jama.2017.19163.

# Appendix 2: Search strategy Medline, Embase

Step 1: Databases Medline, Embase were searched via OVID for the following terms:

| 1 | Point of care test *.ti,ab,kw. |
| --- | --- |
| 2 | POCT .ti,ab,kw. |
| 3 | Near patient test*.ti,ab,kw. |
| 4 | NPT.ti,ab,kw. |
| 5 | 1 or 2 or 3 or 4 |
| 6 | Laboratory.ti,ab,kw. |
| 7 | Cholesterol.ti,ab,kw. |
| 8 | HDL*.ti,ab,kw. |
| 9 | LDL*.ti,ab,kw. |
| 10 | lipid* .ti,ab,kw. |
| 11 | OR/7 – 10 |
| 12 | 5 and 6 and 11 |
| 13 | limit 12 to (english language and humans and yr="2009 -Current") |

Searches were limited to these terms appearing in Title, Abstract, Keywords fields and papers available in English.

Step 2: Databases Medline, Embase were searched via OVID for the following terms:

| 1 | (("Piccolo" OR "Piccolo Xpress" OR "PiccoloXpress" OR "Xpress").af. |
| --- | --- |
| 2 | (("Accutrend Plus " OR "Accutrend" OR "AccutrendPlus").af. |
| 3 | (("Afinion AS100" OR "AfinionAS100" OR "Afinion").af. |
| 4 | (("CardioChek PA" OR "CardioChekPA" OR " CardioChek").af. |
| 5 | (("Cholestech LDX" OR " CholestechLDX" OR "Cholestech").af. |
| 6 | (("Cobas b101" OR " Cobas b 101" OR "b101").af. |
| 7 | "Elemark".af. |
| 8 | OR/1 – 7 |
| 9 | limit 8 to humans (english language and humans and yr="2009 -Current") |

Searches were limited to these terms appearing in All Fields and papers available in English.

# Appendix 3: Eligibility criteria in a PICOS format

| PICOS strategy | Inclusion criteria | Exclusion criteria |
| --- | --- | --- |
| Population | Healthy adults without a pre-existing CVD (from low and middle income countries as well as high income countries*). | Children  Adults with pre exiting CVD (e.g. cardiac patients) |
| Intervention | POCT for cholesterol (TC, HDL-C)  For professional use  As a screening test | POCT for other conditions (glucose, coagulation testing)  Over the counter, single-use disposable total cholesterol measurement kits for self-testing at home. |
| Comparison/control | Laboratory test or another POCT | No comparison  Non-diagnostic comparison  Non-relevant Comparator (clinical judgment, symptom checklists, or imaging) |
| Outcomes | EDTA, ethylenediaminetetraacetic acid; HDL-C, high density lipoprotein cholesterol; LDL-C, low density lipoprotein cholesterol; TC, total cholesterol TG, triglycerides; vs, versus; WB, whole blood  Analytical validity measures  Mean bias (mmol/L), [Limits of agreement], (95% CI), Mean percent bias, ±SD  Clinical validity - diagnostic accuracy measures  Coefficient of variation (%)  Total error (%) | Any other outcome measures |
| Study design | analytical and clinical validity (diagnostic accuracy studies) | clinical utility and health economics and other study designs  qualitative studiers  non-human studies |

PICOS - Population, Intervention, Comparison, Outcome and Study Design

*Added in the limitations

# Appendix 4: QUADAS-2 results

| **Study** | **RISK OF BIAS** | | | | **APPLICABILITY CONCERNS** | | |
| --- | --- | --- | --- | --- | --- | --- | --- |
|  | **PATIENT SELECTION** | **INDEX TEST** | **REFERENCE STANDARD** | **FLOW AND TIMING** | **PATIENT SELECTION** | **INDEX TEST** | **REFERENCE STANDARD** |
| Abbai | ? | ☺ | ? | ☹ | ☺ | ☺ | ☺ |
| Coquiero | ☺ | ☺ | ? | ☺ | ☺ | ☺ | ☺ |
| Kurstjens | ☺ | ☺ | ? | ☹ | ☺ | ☺ | ☺ |
| Maciel | ☺ | ☺ | ? | ☺ | ☺ | ☺ | ☺ |
| Parikh | ☺ | ☺ | ☹ | ☺ | ? | ☺ | ☺ |

☺Low Risk ☹High Risk   ? Unclear Risk

| RISK OF BIAS | APPLICABILITY CONCERNS |
| --- | --- |
| Patient selection | |
| Could the selection of patients have introduced bias?  Was a consecutive or random sample of patients enrolled?  Was a case-control design avoided?  Did the study avoid inappropriate exclusions? | Is there concern that the included patients do not match the review question? |
| Index test | |
| Could the conduct or interpretation of the index test have introduced bias?  Were the index test results interpreted without knowledge of the results of the reference standard?  If a threshold was used, was it pre-specified? | Is there concern that the index test, its conduct, or interpretation differ from the review question? |
| Reference standard | |
| Could the reference standard, its conduct, or its interpretation have introduced bias?  Is the reference standard likely to correctly classify the target condition?  Were the reference standard results interpreted without knowledge of the results of the index test? | Is there concern that the target condition as defined by the reference standard does not match the review question? |
| Flow and timing |  |
| Could the patient flow have introduced bias?  Was there an appropriate interval between index test(s) and reference standard?  Did all patients receive a reference standard?  Did patients receive the same reference standard?  Were all patients included in the analysis? |  |

# Appendix 5: Modified QAREL results

| Study, publication year | Prospective study design? | Was the execution of the index test described in sufficient detail to permit replication of the test? Yes (protocol available), Unclear (no protocol), No. | Was the index test, its conduct, or interpretation aligned with the study aim? | Was a quality control for the index test used? | Was an appropriate reference standard used e.g measurement range, sample type? (E.g. Fasting blood for ref used?) | Was a quality control for the reference standard used? | Is the reference standard the best available reference standard (NCEP/Cholesterol Reference Method Laboratory Network (CRMLN)/ National Health Laboratory Service (NHLS) or similar)? | Time between IT and RS <24 hrs? | Were the samples appropriate to the range of measurement? (High and low ranges included) | Is there sufficient measurement replication (triplicates are minimum to calculate accurately error and bias)? | Is the reporting of error and bias appropriate? (Mean bias, CIs, BA limits of agreement) | Was the statistical analysis performed appropriately? (BA, kappa, regression, sample size, Lin's concordance not t-tests, ANOVA, chi-squared) | Funding by manufacturer? | Were raters blinded to their own prior findings of the test under evaluation? (4) | Were raters blinded to the subjects' disease status or the results of the accepted reference standard for the target disorder (or variable) being evaluated? (5) | Were raters blinded to clinical information that was not intended to form part of the study design or testing procedure? (6) | Were raters blinded to additional cues that are not part of the test? (7) | Was the test evaluated in a sample of subjects who were representative of those to whom the authors intended the results to be applied? (1) | Was the test performed by raters who were representative of those to whom the authors intended the results to be applied? (2) | Was the order of examination varied? (8) |
| --- | --- | --- | --- | --- | --- | --- | --- | --- | --- | --- | --- | --- | --- | --- | --- | --- | --- | --- | --- | --- |
|  | [1] | [2] | [3] | [4] | [5] | [6] | [7] | [8] | [9] | [10] | [11] | [12] | [13] | [14] | [15] | [16] | [17] | [18] | [19] | [20] |
| Barrett, 2014 | Yes | Uncl | Yes | Uncl | No | Uncl | Yes | Yes | Yes | No | Yes | Yes | No | Uncl | Uncl | Uncl | Uncl | Yes | Yes | No |
| Barroso, 2018 | Yes | Yes | Yes | Yes | Yes | Yes | Yes | Yes | Yes | Uncl | Yes | Yes | Yes | Uncl | Uncl | Uncl | Uncl | Yes | Yes | Yes |
| Bastianelli et al, 2017 | Yes | Yes | Yes | Uncl | No | Uncl | Uncl | Uncl | Uncl | No | Yes | Yes | Yes | Uncl | Uncl | Uncl | Uncl | Yes | Yes | No |
| Bolodeoku, 2018 | Yes | Yes | Yes | Uncl | N/A | N/A | No | N/A | No | No | Yes | Yes | Uncl | Uncl | Uncl | Uncl | Uncl | Yes | Uncl | No |
| Bolodeoku, 2019 | Yes | Yes | Yes | Uncl | Yes | No | Yes | Yes | Yes | Yes | Yes | Yes | Uncl | Uncl | Uncl | Uncl | Uncl | Yes | Uncl | No |
| Coquiero, 2013 | Yes | Yes | Yes | No | No | Uncl | Yes | Uncl | Yes | Yes | Yes | Yes | No | Uncl | Uncl | Uncl | Uncl | Yes | Yes | No |
| Donato, 2015 | Yes | Yes | Yes | Uncl | No | Uncl | Yes | Uncl | Yes | No | Yes | Yes | Uncl | Uncl | Uncl | Uncl | Uncl | Yes | Uncl | No |
| dos Santos Ferreira, 2015 | Yes | Yes | Uncl | Yes | Yes | Yes | Yes | Yes | Yes | Yes | Yes | Yes | Yes | Uncl | Uncl | Uncl | Uncl | Yes | Yes | No |
| ICCnet CHSA, 2013 | Yes | Uncl | Yes | Yes | No | Uncl | Yes | Uncl | Uncl | Yes | Yes | Yes | Uncl* | Uncl | Uncl | Uncl | Uncl | Yes | Yes | No |
| Jain et al, 2011 | Yes | Yes | Yes | Yes | No | Yes | Uncl | No | Yes | No | Yes | Yes | Uncl | Uncl | Uncl | Uncl | Uncl | Yes | Yes | No |
| Kurstjens, 2021 | Yes | Yes | Yes | Uncl | No | Yes | Uncl | Uncl | Uncl | No | Yes | Yes | No | Uncl | Uncl | Uncl | Uncl | Yes | Yes | No |
| Maciel, 2019 | Yes | Uncl | Yes | Uncl | No | Uncl | Yes | Uncl | Uncl | No | Yes | Yes | No | Uncl | Uncl | Uncl | Uncl | Yes | Uncl | No |
| Mendez-Gonzalez, 2010 | Yes | Yes | Yes | Yes | Yes | Yes | Yes | Uncl | No | Yes | No | Yes | No* | Uncl | Uncl | Uncl | Uncl | Yes | Yes | No |
| O’Donovan, 2011 | Yes | Yes | Yes | Uncl | Yes | Uncl | Yes | Yes | Uncl | No | Yes | Yes | No | Uncl | Uncl | Uncl | Uncl | Yes | Yes | No |
| Ordonez-Llanos, 2013 | Yes | Uncl | No | Yes | Yes | Yes | Yes | Uncl | Yes | No | Yes | Yes | Yes | Uncl | Uncl | Uncl | Uncl | Yes | Yes | Uncl |
| Parikh, 2009 | Yes | Yes | Yes | Uncl | No | Uncl | Yes | Uncl | Uncl | No | Yes | Yes | No* | Uncl | Uncl | Uncl | Uncl | Yes | Yes | No |
| Park, 2015 | Yes | Yes | Yes | Uncl | No | Uncl | Yes | No | Uncl | No | Yes | Yes | Uncl | Uncl | Uncl | Uncl | Uncl | Yes | Yes | No |
| Scafoglieri, 2012 | Yes | Yes | Yes | Yes | Yes | Yes | Yes | Uncl | No | No | Yes | Yes | Uncl | Yes | Uncl | Uncl | Uncl | Yes | Uncl | No |
| Whitehead, 2013 | Yes | Yes | Yes | Yes | Yes | Yes | Yes | Yes | Yes | No | Yes | Yes | No | Uncl | Uncl | Uncl | Uncl | Yes | Uncl | No |
| Whitehead, 2014 | Yes | Yes | Yes | Yes | Yes | Yes | Uncl | Yes | Yes | No | Yes | Yes | No | Uncl | Uncl | Uncl | Uncl | Yes | Uncl | No |
| Yun, 2019 | Yes | Yes | Yes | No | Yes | No | Uncl | Yes | No | No | Uncl | Yes | Yes | Uncl | Uncl | Uncl | Uncl | Yes | Yes | No |
| Barrett, 2014 | Yes | Uncl | Yes | Uncl | No | Uncl | Yes | Yes | Yes | No | Yes | Yes | No | Uncl | Uncl | Uncl | Uncl | Yes | Yes | No |
| Barroso, 2018 | Yes | Yes | Yes | Yes | Yes | Yes | Yes | Yes | Yes | Uncl | Yes | Yes | Yes | Uncl | Uncl | Uncl | Uncl | Yes | Yes | Yes |
| Bastianelli et al, 2017 | Yes | Yes | Yes | Uncl | No | Uncl | Uncl | Uncl | Uncl | No | Yes | Yes | Yes | Uncl | Uncl | Uncl | Uncl | Yes | Yes | No |
| Bolodeoku, 2018 | Yes | Yes | Yes | Uncl | N/A | N/A | No | N/A | No | No | Yes | Yes | Uncl | Uncl | Uncl | Uncl | Uncl | Yes | Uncl | No |
| Bolodeoku, 2019 | Yes | Yes | Yes | Uncl | Yes | No | Yes | Yes | Yes | Yes | Yes | Yes | Uncl | Uncl | Uncl | Uncl | Uncl | Yes | Uncl | No |

*Uncl - Unclear*

| **Quality Appraisal of Diagnostic Reliability (QAREL) Checklist (Lukas et al., 2010).** | **Questions used in the modified version of QAREL tool** |
| --- | --- |
| Patient selection | |
|  | Prospective study design? (mostly case-control, retrospective) [1] |
| Was the test evaluated in a sample of subjects who were representative of those to whom the authors intended the results to be applied? (1) | same |
| Was the test performed by raters who were representative of those to whom the authors intended the results to be applied? (2) | same |
| Blinding | |
| Were raters blinded to their own prior findings of the test under evaluation? (4) | same |
| Were raters blinded to the subjects' disease status or the results of the accepted reference standard for the target disorder (or variable) being evaluated? (5) | same |
| Were raters blinded to clinical information that was not intended to form part of the study design or testing procedure? (6) | same |
| Were raters blinded to additional cues that are not part of the test? (7) | same |
| Index test | |
|  | Is there sufficient measurement replication (triplicates are minimum to calculate accurately error and bias)?[10] |
|  | Were the samples appropriate to the range of measurement? (High and low ranges included) [9] |
|  | Was a quality control for the index test used? [4] |
| Was the test performed by raters who were representative of those to whom the authors intended the results to be applied? (3) | Was the execution of the index test described in sufficient detail to permit replication of the test? Yes (protocol available), Unclear (no protocol), No. [2] |
| Was the test applied correctly and interpreted appropriately? (10) | Was the index test, its conduct, or interpretation aligned with the study aim? [3] |
| Were appropriate statistical measures of agreement used? (11) | Is the reporting of error and bias appropriate? (Mean bias, CIs, BA limits of agreement) [11] |
|  | Was the statistical analysis performed appropriately? (BA, kappa, regression, sample size, Lin's concordance not t-tests, ANOVA, chi-squared) [12] |
| Reference standard | |
|  | Was an appropriate reference standard used e.g measurement range, sample type? (E.g. Fasting blood for ref used?) [5] |
|  | Was a quality control for the reference standard used? [6] |
|  | Is the reference standard the best available reference standard (NCEP/Cholesterol Reference Method Laboratory Network (CRMLN)/ National Health Laboratory Service (NHLS) or similar)? [7] |
| Was the order of examination varied? (8) | same |
| Time interval | |
| Was the stability (or theoretical stability) of the variable being measured taken into account when determining the suitability of the time interval among repeated measures? (9) | Time between IT and RS <24 hrs? [8] |
| Commercial funding | |
|  | Funding by manufacturer?[13] |

Quality Appraisal of Diagnostic Reliability (QAREL) Checklist (Lukas et al., 2010).

# Appendix 6: NCEP Recommended Performance Criteria for Manufacturers and Clinical Laboratories

| Analyte | Total allowable error (%) | Maximum allowable bias or inaccuracy (%) | Maximum allowable imprecision (CV, %) |
| --- | --- | --- | --- |
| TC [56] | 8.9% | ±3% | 3% |
| HDL-C [57] | 12.8% | ±5% | 4% |
| TG [58] | 14.8% | ±5% | 5% |

Abbreviations: NCEP – National Cholesterol Education Program, TC -Total cholesterol, TG - Triglycerides, HDL-C - High density lipoprotein, CV - Coefficient of variation.

# Appendix 7: Agreement measures between POCTs and the comparator

| Index test POCT name, Manufacturer, Country | Reference | Type of lipid measured | Agreement |
| --- | --- | --- | --- |
| Accutrend Plus, Roche Diagnostics, UK | Barrett [23] | TC ☐  TG ☒  HDL-C ☐  LDL-C ☐  Non-HDL-C ☐ | Passing-Bablok regression  TC: y = 0.98x–0.01, slope = 0.98 [95%[CI]: 0.85-1.15); intercept = -0.01 [-0.051-0.38], r = 0.89, P <0.0001. |
|  | Coquiero [19] | TC ☒  TG ☒  HDL-C ☐  LDL-C ☐  Non-HDL-C ☐ | Lin’s concordance coefficient  TC: r=0.944 (0.905, 0.967)  TG: r=0.990 (0.982,0.994)  Kappa statistic  TC: 0.73  TG: 0.88. |
|  | Kurstjens [20] | TC ☒  TG ☒  HDL-C ☐  LDL-C ☐  Non-HDL-C ☐ | Passing-Bablok regression  TC: y = 1.08x–0.21, slope estimate was 1.08 (95% CI: 1.00-1.19) and the intercept estimate was -0.21 (95% CI: -0.76-0.25), r: 0.94. |
|  | Scafoglieri [24] | TC ☒  TG ☒  HDL-C ☐  LDL-C ☐  Non-HDL-C ☐ | Intraclass correlation coefficient  TC: 0.85, P < 0.001  TG: 0.68, P < 0.001  Weighted kappa  TC: 0.67  TG: 0.50 |
|  | Maciel [26] | TC ☒  TG ☒  HDL-C ☐  LDL-C ☐  Non-HDL-C ☐ | Lin's Concordance Coefficient  [95%CI]  TC: 0.86 [0.74; 0.98], p<0.001  TG: 0.91 [0.84; 0.98], p<0.001  Spearman correlation coefficient  TC: 0.89, p<0.001  TG: 0.4, p<0.001 |
| Cobas b101 System, Roche Diagnostics, Switzerland | Barroso [27] | TC ☒  TG ☐  HDL-C ☒  LDL-C ☐  Non-HDL-C ☐ | Lin's Concordance Coefficient (mmol/L) (95% CI)  Men  TC: 0.0478 [0.0383 – 0.0511]  HDL-C: 0.0506 [0.0489 – 0.0517]  Women  TC: 0.050 (0.0411 – 0.0528)  HDL-C: 0.050 [0.0422 – 0.0528]  Linear regression (r2)  Men  TC: 0.832  HDL-C: 0.869  Women  TC: 0.867  HDL-C: 0.880 |
|  | ICCnet CHSA[28] | TC ☒  TG ☒  HDL-C ☒  LDL-C ☐  Non-HDL-C ☐ | Passing-Bablok regression  TC: y = 0.96x - 0.05, r= 0.981  TG: y = 0.97x + 0.08, r= 0.959  HDL-C: y = 1.23x - 0.31, r= 0.955 |
|  | Ordonez-Llanos/Roche [29] | TC ☒  TG ☒  HDL-C ☒  LDL-C ☐  Non-HDL-C ☐ | Passing-Bablok regression  Capillary whole blood -  TC: y=1.029x -0.175; r = 0.9906  TG: y= 1.014x +0.018; r = 0.9909  HDL-C: y= 0.933x +0.115; r = 0.9748  EDTA whole blood  TC: y=1.00x-0.10, r=0.9930  TG: y=1.00x +0.00, r = 0.9939  HDL-C: y=0.92x+0.10, r=0.9806  EDTA plasma -  TC: y=1.00x -0.11, r= 0.9942  TG: y=0.99x + 0.01, r= 0.9939  HDL-C: y=0.98x + 0.01, r= 0.9817. |
| Afinion AS100, Abbott, USA | Abbai [18] | TC ☒  TG ☒  HDL-C ☒  LDL-C ☒  Non-HDL-C ☐ | Lin's Concordance Coefficient  TC: 0.80  LDL-C: 0.75  HDL: 0.93  TG: 0.99 |
|  | Jain [30] | TC ☒  TG ☒  HDL-C ☒  LDL-C ☐  Non-HDL-C ☐ | Deming Regression  Afinion AS100 vs Cholestech  LDX:  TC: 0.46 + 0.91x.  HDL-C: 0.04 + 1.07x.  TG: 0.05 + 1.02x.  Afinion vs Laboratory:  TC: 0.46 + 0.85x  TG: 0.00 + 1.02x  HDL-C: -0.13 + 1.09x |
| Cholestech LDX, Abbott, USA | Gialamas [34] | TC ☒  TG ☐  HDL-C ☒  LDL-C ☐  Non-HDL-C ☐ | Lin's Concordance Coefficient  TC: 0.85  HDL-C: 0.86  TG: 0.89 |
|  | Jain [35] | TC ☒  TG ☒  HDL-C ☒  LDL-C ☐  Non-HDL-C ☐ | Deming Regression  Cholestech LDX vs lab  TC: -0.62 + 1.07x, r2 =0.83  HDL-C: -0.33 + 1.16x, r2 =0.66  TG -0.00 + 1.01x, r2=0.71  Weighted Kappa = 0.92 |
|  | Motta [36] | TC ☒  TG ☒  HDL-C ☒  LDL-C ☒  Non-HDL-C ☐ | Passing-Bablok regression  TC: y = 0.86x + 0.28, r = 0.98  HDL: y = 1.16x – 0.31, r = 0.93  LDL-C: y = 0.85x + 0.09, r = 0.94  TG: y = 1.10x – 0.07, r = 0.99  Lin's Concordance Coefficient  TC: (<4.0 mmol/L) = 0.84  HDL-C: (>1.0 mmol/L) = 0.89  LDL-C: (<2.5 mmol/L) = 0.88  TG: (<1.5 mmol/L) = 0.96 |
|  | Parikh [38] | TC ☒  TG ☒  HDL-C ☒  LDL-C ☒  Non-HDL-C ☐ | Pearson Correlation Coefficient  TC: r= 0.91  LDL-C: r = 0.88  HDL-C: r = 0.77  TG: r = 0.93  Kappa Statistic  TC: 0.75  LDL-C: 0.75  HDL-C: 0.40  TG: 0.78 |
| CardioChek PA Analyzer, Polymer Technology Systems, USA | dos Santos Ferreira [44] | TC ☒  TG ☒  HDL-C ☒  LDL-C ☐  Non-HDL-C ☐ | Linear regression  Capillary  TC = 9.2+0.92x, r=0.854  HDL-C = 3+1.01x, r=0.936  TG = -4+1.16x, r=0.969  Venous  TC = 11.8+0.87x, r=0.856  HDL-C =5.5+1x, r=0.923  TG = -2.6+1.06x, r=0.953  Capillary vs Venous  TC slope =0.92, r=0.879  HDL-C slope =0.95, r=0.953  TG slope =1.03, r=0.953 |
| Elemark, BBB Tech, South Korea | Yun [47] | TC ☒  TG ☒  HDL-C ☒  LDL-C ☐  Non-HDL-C ☐ | Linear regression  TC: 0.9598x + 4.8881, r = 0.97  TG: 0.985x + 4.489, r = 0.99  HDL-C: 0.9878x -1.3096, r=0.97 |

# Appendix 8: Features and Specifications of the Major Cholesterol POCT Devices.

| POCT brand name, Manufacturer, Country | Available measures | Device type | Measurement range (mmol/L) | Time to results | Specimen volume, specimen type | Quality control (NCEP standard)  ** | Device cost estimate, testing supplies cost estimate |
| --- | --- | --- | --- | --- | --- | --- | --- |
| Accutrend Plus, Roche Diagnostics, Switzerland | Glucose, TC, TG, Lactate | Hand-held device | TC: 3.88 - 7.76  TG: 0.80 – 6.86 | 3 min | 15–40 µL capillary blood | No | £250  £2.88 per test |
| Cobas b101 System, Roche Diagnostics, Switzerland | HbA1c, CRP, TC, TG, HDL-C, LDL-C, TC/HDL-C *, Non-HDL-C *, | Desktop analyser | TC: 1.28 - 12.95  TG: 0.51 - 7.35  HDL-C: 0.39 - 2.59 | / | 19 μL capillary, whole blood or plasma | Yes | £2,992  £8.5 per test |
| Afinion AS100, Abbott, USA | HbA1c, Lipid Panel, ACR, CRP | Desktop analyser | TC: 2.59 - 12.95  TG: 0.51 - 7.35  HDL-C: 0.39 - 2.60 | 3 min | 1.5 µL capillary blood | No | £1,200  £3.50 per test |
| Cholestech LDX, Abbott, USA | TC, TG, HDL-C, LDL-C *, TC/HDL-C *, non-HDL-C * | Desktop analyser | TC: 2.59 - 12.95  TG: 0.51 - 7.35  HDL-C: 0.39 - 2.59 | 5 min | 35-40μL venous blood | No | £1,250  £8.50 per test |
| CardioChek PA Analyzer, Polymer Technology Systems, USA | Glucose, TC, TG, HDL-C. Calculated cholesterol ratios. | Hand-held device | TC: 2.59-10.36  TG: 0.57-5.65  HDL-C: 0.39-2.59 | 2 min - | 15 – 40 µL capillary blood | Yes | £800  £8.50 per test |
| Elemark lipid device, BBB Tech, South Korea | TC, TG, HDL-C, LDL-C * | Hand-held device | IFU not available | 3 min | 5 μL whole blood capillary  or venous | Unknown | $500  per test |

Abbreviations: HbA1c- Haemoglobin A1c, TC -Total cholesterol, TG - Triglycerides, HDL-C - High density lipoprotein cholesterol, LDL-C - Low density lipoprotein cholesterol, non-HDL-C - non high-density lipoprotein cholesterol, VLDL-C - very low density lipoprotein, CRP - C-reactive protein; * Calculated; ** Meets NCEP standard of evidence for bias and precision, Cholesterol Reference Method Laboratory Network (CRMLN) Certified, IFU - Instructions for Use.
