# Supplementary material for: Can we trust published evidence on point-of-care tests for cholesterol? A rapid review
Source: BMJ Open. 2025 Mar 5;15(3):e080726. doi: 10.1136/bmjopen-2023-080726 (PMC11883607; doi:10.1136/bmjopen-2023-080726)
Supplement: Supplementary file 2 [file bmjopen-2023-080726.reviewer_comments.pdf]

PEER REVIEW HISTORY

BMJ Open publishes all reviews undertaken for accepted manuscripts. Reviewers are asked to complete a checklist review form and are provided with free text boxes to elaborate on their assessment. These free text comments are reproduced below.

ARTICLE DETAILS

Title (Provisional)

Can we trust published evidence on point-of-care tests for cholesterol? A rapid review

Authors

Mutepfa, Chikomborero Cynthia; Hicks, Timothy Patrick; Winter, Amanda; Dickinson, Rachel Emma; Williams, Cameron; Harrison, Nick; Chidanyika, Joe; Newton, Julia L.; Jones, William Stephen; Suklan, Jana

---

VERSION 1 - REVIEW

**Reviewer**	**1**
**Name**	**Blais, Joseph**
**Affiliation**	**The University of Hong Kong School of Public Health**
**Date**	**22-Oct-2023**
**COI**	**None to declare.**

Mutepfa and colleagues performed a review of published analytic validity and diagnostic accuracy studies on point-of-care blood lipid testing devices to determine whether they meet the accuracy specifications in the 1995 US NCEP recommendations. As described by the authors, this is an important topic of inquiry for the NHS and for primary health care providers such as pharmacists or family physicians who may offer POCT services. However, I have several concerns that reduce my confidence in the work and I remain uncertain if the current manuscript is in fact original research (systematic review) or a rapid literature review of the available testing devices with the author's expert opinion. For the latter type of review, another venue may be more suitable for this work (e.g., review journal, expert opinion series).

General comments

1. For a review on blood lipid measurement, there is limited consideration of which lipids are of most interest and how are they measured with each device. The abstract lists total cholesterol (TC), low-density lipoprotein cholesterol (LDL-C), high-density lipoprotein cholesterol (HDL-C) and triglycerides, while the introduction specifically mentions use of non-HDL-C (TC minus HDL-C) as the lipid measure used by the QRISK prediction model. If this is the rationale for the review, then TC and HDL-C would be of most interest as they are used to calculate non-HDL-C. Could the authors elaborate on if the POCT devices perform a direct LDL-C measurement or do they calculate LDL-C using Friedwald or another formula,

hence the need for measurement of triglycerides? These are important considerations that do not seem to be considered nor discussed in the manuscript.

2. There appears to be limited consideration of whether the studies testing the POCT test accuracy in the fasting or non-fasted state which may affect the accuracy of the POCT devices. Fasting may affect each component of the lipid panel differently and the accuracy of the POCT devices.
3. Throughout the manuscript the abbreviations used for low-density lipoprotein cholesterol, high-density lipoprotein cholesterol and non-high-density-lipoprotein cholesterol are all missing the C for cholesterol (e.g., LDL-C). This is confusing since one needs to distinguish the LDL particle and the LDL cholesterol content of the particle (what is measured or calculated in practice).
4. The review was not pre-registered, and no protocol was submitted by the authors. This increases the risk of bias and lowers my certainty of the systematic nature of the review. Therefore, I feel the work may be best positioned as an expert opinion literature review.
5. The submitted PRISMA checklist is not the latest version (2020). Authors should use the latest PRISMA checklist for the manuscript and the abstract checklist for the abstract. Also, for Figure 1, they should follow updated guidance for the flow diagram, and include details about the specific sources and number of records identified from these sources. See PRISMA website: http://www.prisma-statement.org/PRISMAStatement/
6. I suggest the authors remove the word impartial from the manuscript. I do not expect an impartial review, but rather a clear, transparent, and systematic process that is well described so that the work could reasonably be reproduced. Authors must always use their judgements throughout the review process and when judging the quality of the included studies. Although the evidence has been gathered in an overview, I think the manuscript fails to give a solid conclusion or recommendation to the reader. If 1-2 POCT devices had to be recommended to the NHS, based on their accuracy, which ones would the author's select?

Abstract

1. If publishing as a systematic review, I would encourage the authors to follow the PRISMA abstract guidelines. Some additional details such as the database search dates, the tools used to assess quality, and the key study outcomes are needed.
2. I don't think it is valid to claim a strong linear relationship for all POCT devices.
3. Some of the statements in the conclusion would be best suited for the results, such as "The majority of evidence focused on three POCTs (Cholestech LDX, CardioChek PA, Accutrend Plus)."

Methods

1. The ROBINS-I tool has been miscited and not applied in the review. This risk of bias tool is not appropriate for the types of studies (analytical and diagnostic accuracy) included in the review. The authors should not modify ROBINs-I. I think they can use their own risk of bias tool for analytic validity studies, but this should be grounded in some theory or evidence for these studies. The proposed tool in Table 1 appears to assess the overall quality (certainty) of the study rather than the internal validity which ROBINS-I is designed to assess.

   Erik Igelström, Mhairi Campbell, Peter Craig, Srinivasa Vittal Katikireddi. Cochrane's risk of bias tool for non-randomized studies (ROBINS-I) is frequently misapplied: A methodological systematic review. Journal of Clinical Epidemiology. Volume 140; 2021. Pages 22-32. https://doi.org/10.1016/j.jclinepi.2021.08.022

2. Was a search of any grey literature preformed and did the authors contact manufacturers or study authors?
3. Please clearly state the outcomes of most interest in the review. For example, is the review focused on total cholesterol and HDL-cholesterol, to calculated non-HDL cholesterol? Is it all components of the standard lipid panel? It seems to me that the focus is on total cholesterol accuracy and precision based on the table in Appendix 5.
4. Eligibility criteria as listed in Appendix 2 and 3 can be combined into a single table that includes all the eligibility criteria in the order that they were applied when assessing a study and the exclusion coding reason.

Results

1. Figure 1 should use the latest PRISMA flowchart style and report the number of records identified from each database.
2. Although the risk of bias in the included studies is important, I think the summary of the evidence for each device in Appendix 5 should be the focus of the results and included as a Table in the main text.
3. Page 9, lines 20-34, there seems to be a formatting problem with duplicate text?
4. Although the authors state the focus of the review is on devices used in primary care, it seems many of the studies were conduct in secondary care (Appendix 6). Could they clarify why these studies where included, and what is the definition of secondary care that the authors used?
5. Would the results be better structured by focusing on each analyte or interest or perhaps each of the POCT devices?
6. I feel that there is no clear result as to which devices are the most accurate and precise and could be practically deployed for use in community settings.

Discussion

1. Suggest the discussion includes 3-5 paragraphs that focus on the subheadings the authors have used.
2. As stated earlier, I would like to see the implications for policy and practice expanded so that we have a clear recommendation based on the review's objective. Which device(s) does the best available evidence suggest are the most accurate and precise for each analyte? What did we learn from the review that decision makers need to consider when selecting a device? The current paragraph talks about the market and transitioning away from CE which isn't clearly linked to the findings of the review.
3. Other limitations included not contacting manufacturers, other unpublished sources, only searching two databases, restricting to English language (some devices are from South Korea and Taiwan, so there may be literature in Korean and Chinese for these POCT), and perhaps heterogeneity of the included study populations (primary care, secondary care, pregnant women, HIV infected, healthy volunteers etc).
4. The authors should narratively synthesize the evidence, but no quantitative synthesis with meta-analysis was performed (p. 13, line 20).

Reviewer **2**

Name **Bell, Katy**

Affiliation **The University of Sydney Faculty of Medicine and Health, School of Public Health**

Date **21-Dec-2023**

COI **None**

Thank you for the opportunity to review this rapid review of POCT for cholesterol. I have some suggestions for how it might be improved.

1. ROBINS-I is not the correct ROB tool to use for analytical validity studies. QAREL is much more appropriate for these types of studies: Lucas, Macaskill, Irwig, Bogduk. The development of a quality appraisal tool for studies of diagnostic reliability (QAREL), Journal of Clinical Epidemiology,2010; 63: 854-861, https://doi.org/10.1016/j.jclinepi.2009.10.002

The adjustments made to ROBINS-I are good, but tend to have created quite a different tool to ROBINS-I, and this tool has not been validated/used in other reviews. Please see if you can incorporate the key elements of QAREL into your ROB. QAREL also aligns better with QUADAS-2 (which you appropriately use for the clinical validity studies).

2. This recently published reporting checklist may also have relevant elements to assess the studies included in your review: Loh, Tze Ping, Cooke, Brian R., Tran, Thi Chi Mai, Markus, Corey, Zakaria, Rosita, Ho, Chung Shun, Theodorsson, Elvar, Greaves, Ronda F. and on behalf of the IFCC Working Group on Method Evaluation Protocols (WG-MEP),. "The LEAP checklist for laboratory evaluation and analytical performance characteristics reporting of clinical measurement procedures" Clinical Chemistry and Laboratory Medicine (CCLM), 2023. https://doi.org/10.1515/cclm-2023-0933

3. At the start of the results, you should provide a description of the selection of studies in words, as well as referring to the PRISMA diagram. You also need to provide an overall description of the included studies, including countries, years, % female, mean age, study population (e.g. population based vs clinical population), etc.

4. QUADAS-2 ratings – I am a bit surprised that there was no concern about patient selection . Were the study populations actually representative of the target populations/use? Can you provide the applicability ratings as well as the risk of bias ones.

5. Results are not described in nearly enough detail. You need to describe results in the text, not just refer to Appendix tables. What were the key findings? The results section needs to be substantially longer.

6. Unclear which NCEP thresholds are referred to on page 11, line 60. I was expecting to read results on precision, concordance (for analytical validity studies), and sensitivity, specificity, AUROC curve and predictive values (for clinical validity studies).

7. Most of the Discussion has results that have not been reported in the Results section.

8. What do you mean by focal POCT on page 14, line 38 - index POCT (index test)?

9. PRISMA -DTA should be used rather than PRISMA for the reporting guideline.

10. PICOS strategy reported in Appendix 3 – you haven't describe the population for the inclusion/exclusion criteria. Instead you have described tests under the population heading. Also for the clinical validity outcomes, you should specify whether there were inclusion/exclusion criteria related to reference standard.

VERSION 1 - AUTHOR RESPONSE

Responses to Reviewer #1

Reviewer: 1
Dr. Joseph Blais, The University of Hong Kong School of Public Health

Mutepfa and colleagues performed a review of published analytic validity and diagnostic accuracy studies on point-of-care blood lipid testing devices to determine whether they meet the accuracy specifications in the 1995 US NCEP recommendations. As described by the authors, this is an important topic of inquiry for the NHS and for primary health care providers such as pharmacists or family physicians who may offer POCT services. However, I have several concerns that reduce my confidence in the work and I remain uncertain if the current manuscript is in fact original research (systematic review) or a rapid literature review of the available testing devices with the author's expert opinion. For the latter type of review, another venue may be more suitable for this work (e.g., review journal, expert opinion series).

We are inclined to call it a rapid review, which is still systematic based on Cochrane's definition. Rapid because we are using two/three databases. Our aim here was to summarize objective measures reported in selected studies, not providing a subjective perspective.

General comments

1. For a review on blood lipid measurement, there is limited consideration of which lipids are of most interest and how are they measured with each device.	Citing National Guidelines.<br>- NHS Lipid programme

The abstract lists total cholesterol (TC), lowdensity lipoprotein cholesterol (LDL-C), highdensity lipoprotein cholesterol (HDL-C) and triglycerides, while the introduction specifically mentions use of non-HDL-C (TC minus HDLC) as the lipid measure used by the QRISK prediction model. If this is the rationale for the review, then TC and HDL-C would be of most interest as they are used to calculate non-HDL-C. Could the authors elaborate on if the POCT devices perform a direct LDL-C measurement or do they calculate LDL-C using Friedwald or another formula, hence the need for measurement of triglycerides?	See Appendix 5<br>This information is provided in the "Available measures" column, if LDL-C is reported that means that POCT devices performs a direct LDLC measurement.
These are important considerations that do not seem to be considered nor discussed in the manuscript.	This has now been added to the discussion.
2. There appears to be limited consideration of whether the studies testing the POCT test accuracy in the fasting or non-fasted state which may affect the accuracy of the POCT devices.	See Table 1<br>The information about the fasted or non-fasted<br>state is described in the Setting/Patient population column.
Fasting may affect each component of the lipid panel differently and the accuracy of the POCT devices.	Guidance produced by the National Institute for Health and Care Excellence (NICE) recommends measurement of non-fasting blood samples, see Background, pg.4
3. Throughout the manuscript the abbreviations used for low-density lipoprotein cholesterol, high-density lipoprotein cholesterol and non-high-density-lipoprotein cholesterol are all missing the C for cholesterol (e.g., LDLC). This is confusing since one needs to distinguish the LDL particle and the LDL cholesterol content of the particle (what is measured or calculated in practice).	This has been amended throughout the manuscript.
4. The review was not pre-registered, and no protocol was submitted by the authors. This increases the risk of bias and lowers my certainty of the systematic nature of the review. Therefore, I feel the work may be best positioned as an expert opinion literature review.	The protocol was developed for internal purposes and was aligned with the requirements of the AHSN which commissioned this piece of work.

5. The submitted PRISMA checklist is not the latest version (2020). Authors should use the latest PRISMA checklist for the manuscript and the abstract checklist for the abstract. Also, for Figure 1, they should follow updated guidance for the flow diagram, and include details about the specific sources and number of records identified from these sources. See PRISMA website: http://www.prismastatement.org/PRISMAStatement/	We have updated the PRISMA to the more appropriate PRISM -DTA (as suggested by the other reviewer).<br><br>PRISMA (prisma-statement.org)
6. I suggest the authors remove the word impartial from the manuscript. I do not expect an impartial review, but rather a clear, transparent, and systematic process that is well described so that the work could reasonably be reproduced. Authors must always use their judgements throughout the review process and when judging the quality of the included studies.	We have removed the word impartial from the manuscript.
Although the evidence has been gathered in an overview, I think the manuscript fails to give a solid conclusion or recommendation to the reader. If 1-2 POCT devices had to be recommended to the NHS, based on their accuracy, which ones would the author's select?	This has been added to the a Abstract and Conclusion Recommendations added.

Abstract

1. If publishing as a systematic review, I would encourage the authors to follow the PRISMA abstract guidelines. Some additional details such as the database search dates, the tools used to assess quality, and the key study outcomes are needed.	This has been utilised.
2. I don't think it is valid to claim a strong linear relationship for all POCT devices.	This statement has been removed.
3. Some of the statements in the conclusion would be best suited for the results, such as "The majority of evidence focused on three POCTs (Cholestech LDX, CardioChek PA, Accutrend Plus)."	The conclusion statements have been amended.

Methods

1. The ROBINS-I tool has been miscited and not applied in the review. This risk of bias tool is not appropriate for the types of studies	Our tool was developed by clinical scientists with expertise in laboratory validation and designed to assess quality.

(analytical and diagnostic accuracy) included in the review. The authors should not modify	
ROBINs-I. I think they can use their own risk of bias tool for analytic validity studies, but this should be grounded in some theory or evidence for these studies. The proposed tool in Table 1 appears to assess the overall quality (certainty) of the study rather than the internal validity which ROBINS-I is designed to assess. Erik Igelström, Mhairi Campbell, Peter Craig, Srinivasa Vittal Katikireddi. Cochrane's risk of bias tool for non-randomized studies (ROBINS-I) is frequently misapplied: A methodological systematic review. Journal of Clinical Epidemiology. Volume 140; 2021. Pages 22-32. https://doi.org/10.1016/j.jclinepi.2021.08.022	
2. Was a search of any grey literature preformed and did the authors contact manufacturers or study authors?	Grey literature was searched on Google scholar and hand searches of systematic review references.<br>Study authors and manufacturers were not contacted and this has been noted as a limitation in our discussion.
3. Please clearly state the outcomes of most interest in the review. For example, is the review focused on total cholesterol and HDLcholesterol, to calculated non-HDL cholesterol? Is it all components of the standard lipid panel? It seems to me that the focus is on total cholesterol accuracy and precision based on the table in Appendix 5.	The review focuses on TC, TG, HDL-C and non-HDL-C based on National Guidance for Lipid Management for Primary and Secondary Prevention of CVD.<br><br>The columns relating to TC measurements have been deleted
4. Eligibility criteria as listed in Appendix 2 and 3 can be combined into a single table that includes all the eligibility criteria in the order that they were applied when assessing a study and the exclusion coding reason.	These have been merged into one in Appendix 2.

Results

1. Figure 1 should use the latest PRISMA flowchart style and report the number of records identified from each database.	We have now used the newest template for the PRISMA flowchart.

2. Although the risk of bias in the included studies is important, I think the summary of the evidence for each device in Appendix 5 should be the focus of the results and included as a Table in the main text.	We have moved the table from Appendix 5 to Tables 2 and 3.
3. Page 9, lines 20-34, there seems to be a formatting problem with duplicate text?	This has been resolved.
4. Although the authors state the focus of the review is on devices used in primary care, it seems many of the studies were conduct in secondary care (Appendix 6). Could they clarify	We acknowledge that several studies in our review were conducted in secondary care settings, however the decision to include studies conducted in secondary care is grounded like evidence generation for medical
why these studies where included, and what is the definition of secondary care that the authors used?	tests, including point-of-care testing (POCT). Traditionally, evidence for medical tests is first generated in tertiary or secondary care settings and then transferred across diverse healthcare settings alongside feasibility studies. Due to factors such as the availability of specialized equipment for comparative purposes, highly skilled and specialised healthcare professionals and higher prevalence of conditions, and other reasons analytical validity and diagnostic accuracy studies are rarely run in primary or community care.
5. Would the results be better structured by focusing on each analyte of interest or perhaps each of the POCT devices?	The results are structured focusing on POCT devices.
6. I feel that there is no clear result as to which devices are the most accurate and precise and could be practically deployed for use in community settings.	A summary of the device performance against NCEP standards has now been provided and a recommendation added based off this.

Discussion

1. Suggest the discussion includes 3-5 paragraphs that focus on the subheadings the authors have used.	We have reduced this to 5 paragraphs.

2. As stated earlier, I would like to see the implications for policy and practice expanded so that we have a clear recommendation based on the review's objective. Which device(s) does the best available evidence suggest are the most accurate and precise for each analyte? What did we learn from the review that decision makers need to consider when selecting a device? The current paragraph talks about the market and transitioning away from CE which isn't clearly linked to the findings of the review.	We have now changed this paragraph to denote the most appropriate device to be selected.
3. Other limitations included not contacting manufacturers, other unpublished sources, only searching two databases, restricting to English language (some devices are from South Korea and Taiwan, so there may be literature in Korean and Chinese for these POCT), and perhaps heterogeneity of the included study populations (primary care, secondary care, pregnant women, HIV infected, healthy volunteers etc).	Thank you so much for pointing these out, we have added them to the weaknesses of the study.
4. The authors should narratively synthesize the evidence, but no quantitative synthesis with meta-analysis was performed (p. 13, line 20).	This is now included in the results.

Responses to Reviewer #2

Reviewer: 2
Dr. Katy Bell, The University of Sydney Faculty of Medicine and Health

1. ROBINS-I is not the correct ROB tool to use for analytical validity studies. QAREL is much more appropriate for these types of studies: Lucas, Macaskill, Irwig, Bogduk. The development of a quality appraisal tool for studies of diagnostic reliability (QAREL), Journal of Clinical Epidemiology,2010; 63: 854-861, https://doi.org/10.1016/j.jclinepi.2009.10.002 The adjustments made to ROBINS-I are good, but tend to have created quite a different tool to ROBINS-I, and this tool has not been validated/used in other reviews. Please see if you can incorporate the key elements of QAREL into your ROB. QAREL also aligns better with QUADAS-2 (which you appropriately use for the clinical validity studies).	QAREL is more appropriate for reliability studies, however most of the studies in our analysis did not meet this criteria, so we have not included this.

2. This recently published reporting checklist may also have relevant elements to assess the studies included in your review: Loh, Tze Ping, Cooke, Brian R., Tran, Thi Chi Mai, Markus, Corey, Zakaria, Rosita, Ho, Chung Shun, Theodorsson, Elvar, Greaves, Ronda F. and on behalf of the IFCC Working Group on Method Evaluation Protocols (WG-MEP),. "The LEAP checklist for laboratory evaluation and analytical performance characteristics reporting of clinical measurement procedures" Clinical Chemistry and Laboratory Medicine (CCLM), 2023. https://doi.org/10.1515/cclm-2023-0933	Although the LEAP checklist was appropriate for analysing the reporting of analytical validity studies, we found it difficult to translate to quality assessment and have not utilised this.
3. At the start of the results, you should provide a description of the selection of studies in words, as well as referring to the PRISMA diagram.	We have added this under the subheading "Studies selection".
You also need to provide an overall description of the included studies, including countries, years, % female, mean age, study population (e.g. population based vs clinical population), etc.	We have added this under the subheading "Characteristics of the included studies".
4. QUADAS-2 ratings – I am a bit surprised that there was no concern about patient selection. Were the study populations representative of the target populations/use? Can you provide	
the applicability ratings as well as the risk of bias ones.	
5. Results are not described in nearly enough detail. You need to describe results in the text, not just refer to Appendix tables. What were the key findings? The results section needs to be substantially longer.	The results for the seven POCTs featured were moved to the results section from the Appendix.
6. Unclear which NCEP thresholds are referred to on page 11, line 60. I was expecting to read results on precision, concordance (for analytical validity studies), and sensitivity, specificity, AUROC curve and predictive values (for clinical validity studies).	The NCEP standards have been clarified.
7. Most of the Discussion has results that have not been reported in the Results section.	The results for the seven POCTs featured were move to the results from the Appendix.

8. What do you mean by focal POCT on page 14, line 38 - index POCT (index test)?	What we mean by focal is the index test of interest, which can be compared either to a lab standard of care (as a comparator test) or another POC test (especially if the study does not have access to the lab).
9. PRISMA -DTA should be used rather than PRISMA for the reporting guideline.	Thank you for pointing this out, we have now transferred to use the PRISMA -DTA template, see Appendix 1.
10. PICOS strategy reported in Appendix 3 – you haven't described the population for the inclusion/exclusion criteria. Instead, you have described tests under the population heading.	This has now been amended. Our inclusion criteria were adult population and we excluded children.
Also for the clinical validity outcomes, you should specify whether there were inclusion/exclusion criteria related to reference standard.	We have added this in the PICOS strategy reported in Appendix 3.

VERSION 2 - REVIEW

Reviewer 1

Name Blais, Joseph

Affiliation The University of Hong Kong School of Public Health

Date 19-Apr-2024

COI None to declare

Good job revising the manuscript. The revisions have very much improved the work and I commend the authors for their efforts to navigate through this heterogenous body of evidence.

I have a few minor points that should be addressed:

- Table 2: First column is missing the name of a POCT test

- The manuscript should be thoroughly proofread to correct some minor errors such as the consistent use of abbreviations (e.g., page 2, line 40) and formatting. There also seems to be a problem with missing references/links in the proof that I am viewing (e.g., page 5, line 12, 23 and 30).

Reviewer 2

Name Bell, Katy

Affiliation **The University of Sydney Faculty of Medicine and Health, School of Public Health**

Date **02-Aug-2024**

COI **none**

Thank you for your responses, but many of my comments have not been addressed.

1. At the end of the Introduction, please include a well structured specific research question for the review, in "PIT" format as recommended by the new Cochrane guidelines: "PIT for test accuracy questions, where the three letters refer to population, index test(s) and target condition. P describes the persons for whom the test is considered, and may include the healthcare setting and tests done prior to using the index test. We prefer 'population' rather than 'patients', because tests may also be used in asymptomatic persons, as in population screening programmes. I describes the index test(s). T describes the target condition."

2. Risk of bias assessment for Analytic validity studies. I disagree that there are no suitable tools available. I note that you decided that you could not use elements from the LEAP checklist, or the QAREL tool. Perhaps you could look at the tools used for genetic tests – for example see: https://www.gimjournal.org/article/S1098-3600(21)02131-6/fulltext#t0025 .

3. You need to not just respond to me about what you mean by focal POCT test, but make changes to the manuscript to make it clear that this is the index test of interest.

4. PICOS strategy – you need to be more specific for the population than just stating it was adults. What are the clinical settings that you were interested in? Was it for healthy adults (as a screening test) or for people already identified as high risk of CVD? Low and middle income countries as well as high income countries? Outcomes – need to state the specific measures, not just repeating the study design.

5. QUADAS-2 ratings. You have provided no response to my comment. Can you please provide more details on patient selection processes, and how closely the study population matches the intended clinical use population. You should justify why you had little or no concerns over patient selection or applicability (note that applicability is about how applicable is the evidence to your review question – i.e. the intended use population, not to the primary study's aims as currently stated in the manuscript). And instead of just identifying studies by the author's name in this section, provide some context for the reader by identifying which POCT the study was evaluating. You also need to state the ROB criteria in detail for each of the domains as a footnote for Appendix 4.

6. Section 3.2, Results. You still have not included key results in section 3.2 about the number of records you retrieved, number of full texts screened before you decided to include 28 studies. And what were the number of patients, samples included for each of the 7 POCTs?

7. Table 1. You need to also include the reference test in this table. Comparator refers to another test under evaluation not the reference test. See: https://journals.lww.com/ijebh/fulltext/2015/09000/diagnostic_test_accuracy__methods_for_systematic.7.aspx#:~:text=Systematic%20reviews%20of%20diagnostic%20test%20accuracy%20are%20used%20to%20provide,in%20findings%20between%20the%20studies.

8. Section 3.3, Results. This is titled Accuracy of the PCOTs, but it appears to also include analytic validity results. This section lacks structure and needs to be edited to make it more coherent, so the reader is able to determine what the key results are. Can you summarise for each PCOT, the analytical validity (including bias, precision, CV, total error), and the clinical validity (paired sensitivity and specificity estimates, AUROC curve (c-statistic) and predictive values. Can you construct a bivariate forrest plot or a SROC plot to display the paired sens/spec estimates for the different POCTs?

9. There appears to be stray text included at the end of the Results section and start of the Discussion that is highlighted. Text in the Discussion appears to be badly formatted with different colours and fonts. Please address these issues.

10. Discussion. You need to actually state the principal findings in 4.1 – did you find that the POCTs were fit for purpose? What was the take home finding for the analytical validity and clinical validity?

VERSION 2 - AUTHOR RESPONSE

Responses to Reviewer #1

Reviewer: 1
Dr. Joseph Blais, The University of Hong Kong School of Public Health

Reviewer #1: General comments

Good job revising the manuscript. The revisions have very much improved the work and I commend the authors for their efforts to navigate through this heterogenous body of evidence.

Reviewer #1: Specific comments

I have a few minor points that should be addressed:

- Table 2: First column is missing the name of a POCT test	We have resolved this and added the name in the cell: Afinion AS100, Abbott, USA.
- The manuscript should be thoroughly proofread to correct some minor errors such as the consistent use of abbreviations (e.g., page 2, line 40) and formatting. There also seems to be a problem with missing references/links in the proof that I am viewing (e.g., page 5, line 12, 23 and 30).	We send the manuscript for proofreading.<br>We have resolved the issue with missing references/links.

Responses to Reviewer #2

Reviewer: 2
Dr. Katy Bell, The University of Sydney Faculty of Medicine and Health

Reviewer #2: General comments

Thank you for your responses, but many of my comments have not been addressed.

1. At the end of the Introduction, please include a well-structured specific research question for the review, in "PIT" format as recommended by the new Cochrane guidelines: "PIT for test accuracy questions, where the three letters refer to population, index test(s) and target condition. P describes the persons for whom the test is considered, and may include the healthcare setting and tests done prior to using the index test. We prefer 'population' rather than 'patients', because tests may also be used in asymptomatic persons, as in population screening programmes. I describes the index test(s). T describes the target condition."	We have added the specific research question in the Background section which reads "Our research questions were to determine what evidence is available about the performance of the POCTs that measure cholesterol in adults compared to current reference standards?". Furthermore, we described the eligibility criteria for this review using Population, Intervention, Comparison, Outcome and Study Design (PICOS) as reported in Appendix 3.<br><br>Our research question was to determine what evidence is available about the performance of the POCTs that could be used in primary care settings to measure cholesterol in adults aged between 40 and 74 years (without pre-existing CVD) compared to current reference standards?
2. Risk of bias assessment for Analytic validity studies. I disagree that there are no suitable tools available. I note that you decided that you could not use elements from the LEAP checklist, or the QAREL tool. Perhaps you could look at the tools used for genetic tests – for example see:<br>*Systematic reviews of diagnostic test accuracy are used to provide a summary of test performance based on all available evidence, evaluate the quality of published studies, and account for variation in findings between the studies*<br>https://www.gimjournal.org/article/S1098-3600(21)02131-6/fulltext#t0025 .<br><br>Round 1 - comment<br>ROBINS-I is not the correct ROB tool to use for analytical validity studies. QAREL is much more appropriate for these types of studies: Lucas, Macaskill, Irwig, Bogduk. The development of a quality appraisal tool for studies of diagnostic reliability (QAREL), Journal of Clinical Epidemiology,2010; 63: 854-861, https://doi.org/10.1016/j.jclinepi.2009.10.002	Initially we developed a proprietary tool for RoB of analytical validity studies, which we wanted to publish as a separate/subsequent paper to address the risk of bias assessment tools available for Analytic validity studies. Given the time pressure and a clear rationale that we need to report RoB in this paper, we ecided to use a modified quality appraisal tool for studies of diagnostic reliability (QAREL).

The adjustments made to ROBINS-I are good, but tend to have created quite a different tool to ROBINS-I, and this tool has not been validated/used in other reviews. Please see if you can incorporate the key elements of QAREL into your ROB. QAREL also aligns better with QUADAS-2 (which you appropriately use for the clinical validity studies).<br><br>Round 1 - response<br>QAREL is more appropriate for reliability studies, however most of the studies in our analysis did not meet this criteria, so we have not included this.<br><br>Round 1 - comment<br>This recently published reporting checklist may also have relevant elements to assess the studies included in your review: Loh, Tze Ping, Cooke, Brian R., Tran, Thi Chi Mai, Markus, Corey, Zakaria, Rosita, Ho, Chung Shun, Theodorsson, Elvar, Greaves, Ronda F. and on behalf of the IFCC Working Group on Method Evaluation Protocols (WG-MEP),. "The LEAP checklist for laboratory evaluation and analytical performance characteristics reporting of clinical measurement procedures" Clinical Chemistry and Laboratory Medicine (CCLM), 2023. https://doi.org/10.1515/cclm-2023-0933<br><br>Round 1 - response<br>Although the LEAP checklist was appropriate for analysing the reporting of analytical validity studies, we found it difficult to translate to quality assessment and have not utilised this.	
3. You need to not just respond to me about what you mean by focal POCT test, but make changes to the manuscript to make it clear that this is the index test of interest.<br><br>Round 1 - comment<br>What do you mean by focal POCT on page 14, line 38 - index POCT (index test)?<br><br>Round 1 - response<br>What we mean by focal is the index test of interest, which can be compared either to a lab standard of care (as a comparator test) or	Thank you for clarifying your point, we have now made changes to the text to make it clearer to the reader that this is the index test of interest.

another POC test (especially if the study does not have access to the lab).	
4. PICOS strategy – you need to be more specific for the population than just stating it was adults. What are the clinical settings that you were interested in? Was it for healthy adults (as a screening test) or for people already identified as high risk of CVD? Low and middle income countries as well as high income countries? Outcomes – need to state the specific measures, not just repeating the study design.<br><br>Round 1 - comments<br>PICOS strategy reported in Appendix 3 – you haven't described the population for the inclusion/exclusion criteria. Instead, you have described tests under the population heading. Also for the clinical validity outcomes, you should specify whether there were inclusion/exclusion criteria related to reference standard.<br><br>Round 1 - response<br>This has now been amended. Our inclusion criteria were adult population and we excluded children.<br>We have added this in the PICOS strategy reported in Appendix 3.	We have now extended the table of characteristics to include this information.
5. QUADAS-2 ratings. You have provided no response to my comment. Can you please provide more details on patient selection processes, and how closely the study population matches the intended clinical use population. You should justify why you had little or no concerns over patient selection or applicability (note that applicability is about how applicable is the evidence to your review question – i.e. the intended use population, not to the primary study's aims as currently stated in the manuscript). And instead of just identifying studies by the author's name in this section, provide some context for the reader by identifying which POCT the study was evaluating. You also need to state the ROB criteria in detail for each of the domains as a footnote for Appendix 4.	JUSTIFY how closely the study population matches the intended clinical use population<br>- we justified why we had little or no concerns over patient selection or applicability<br>- We added to the author's name also the name of the POCT the study was evaluating<br>- We added QUADAS-2 domains as a footnote in the Appendix.<br><br>We excluded studies that recruited patients with the existing CVD.<br><br>For the analytical validity studies we now included a risk of bias table to the appendices, including the question "Was the test evaluated in a sample of subjects who were representative of those to whom the authors

Round 1 - comment<br>QUADAS-2 ratings – I am a bit surprised that there was no concern about patient selection. Were the study populations representative of the target populations/use? Can you provide the applicability ratings as well as the risk of bias ones.<br><br>Round 1 – response<br>This was not addressed in the previous round.	intended the results to be applied?". All analytical validity studies evaluated subjects that were representative of our target population, which is adults without preexisting CVDs.<br><br>Since QUADAS-2 is assessing the patient selection based on three questions:<br>• random or consecutive patient selection?<br>• case-control design avoided?<br>• Did the study avoid inappropriate exclusions?<br>we extracted additional information to confirm that in all diagnostic accuracy studies they evaluated subjects that were representative of our target population.<br><br>In terms of patient selection we know that random or consecutive patient selection was used in four out of five diagnostic accuracy studies; and the case control design was avoided in all the studies.<br><br>All patients had an equal chance of being recruited thus there were no concerns in patient selection. People younger than 18 or with known cardiovascular problems were excluded as their results could be outliers.<br><br>In terms of patient selection we know that random or consecutive patient selection was used in four out of five diagnostic accuracy studies; and the case control design was avoided in all the studies.
6. Section 3.2, Results. You still have not included key results in section 3.2 about the number of records you retrieved, number of full texts screened before you decided to include 28 studies. And what were the number of patients, samples included for each of the 7 POCTs?<br><br>Round 1 - comment<br>At the start of the results, you should provide a description of the selection of studies in words, as well as referring to the PRISMA diagram. You also need to provide an overall description of the included studies, including countries, years, % female, mean age, study population	We have included in the results section addition information - the number of records retrieved, number of full texts screened, number of patients, samples included for each of the POCTs.

(e.g. population based vs clinical population), etc.<br><br>Round 1 – response<br>We have added this under the subheading "Studies selection".<br>We have added this under the subheading "Characteristics of the included studies".	
7. Table 1. You need to also include the reference test in this table. Comparator refers to another test under evaluation not the reference test. See: https://journals.lww.com/ijebh/fulltext/2015/09000/diagnostic_test_accuracy__methods_for_systematic.7.aspx#:~:text=Systematic%20reviews%20of%20diagnostic%20test%20accuracy%20are%20used%20to%20provide,in%20findings%20between%20the%20studies	Table 1: we have renamed the first column Index test POCT name, Manufacturer, Country since we are interested in the index test is the diagnostic test whose accuracy is being investigated.<br><br>In the fourth column we have replaced the term Comparator with "Reference standard" where we also specify the sample type used in the analysis, since we use this term in text. It must be noted however that by reference standard we don`t necessarily mean the 'gold standard' or best test currently available for the diagnosis of the condition of interest, but rather a laboratory or in few cases another POCT test against which the results of the index test are compared against.
8. Section 3.3, Results. This is titled Accuracy of the PCOTs, but it appears to also include analytic validity results.<br><br>This section lacks structure and needs to be edited to make it more coherent, so the reader is able to determine what the key results are.<br><br>Can you summarise for each POCT, the analytical validity (including bias, precision, CV, total error), and the clinical validity (paired sensitivity and specificity estimates, AUROC curve (c-statistic) and predictive values. Can you construct a bivariate forrest plot or a SROC plot to display the paired sens/spec estimates for the different POCTs?	The subtitle has now been changed to "Analytical validity and accuracy of the POCTs"<br><br>• For clarity, we have added a sentence above the summation of results for each POCT.<br>• All estimates that are available in the studies have been included in the table and summarised in the text.<br>• We did not have sufficient data for all POCTs to conduct meta-analysis, thus we only provide a narrative synthesis as per Higgins JPT, Thomas J, Chandler J, Cumpston M, Li T, Page MJ, Welch VA (editors). *Cochrane Handbook for Systematic Reviews of Interventions* version 6.5 (updated August 2024). Cochrane, 2024. Available from www.training.cochrane.org/handbook.<br>• We acknowledge the reviewers' suggestion to construct a bivariate forest plot or SROC plot to illustrate paired sensitivity and specificity estimates for POCTs. However, based

	on the available data (there are data for three tests but only one test is supported by more than one study): for the two tests with single-study data, there is no variability in sensitivity and specificity estimates to display, which relies on variation/ pooled estimates across studies to summarize diagnostic accuracy and visualize the trade-off between sensitivity and specificity. Including tests with data from only a single study alongside a test supported by multiple studies may create a misleading impression of the evidence base. It may appear as though there is comparable support for all three tests, whereas the robustness of the data is significantly different.
9. There appears to be stray text included at the end of the Results section and start of the Discussion that is highlighted. Text in the Discussion appears to be badly formatted with different colours and fonts. Please address these issues.	Thank you for noticing this, this has now been resolved.
10. Discussion. You need to actually state the principal findings in 4.1 – did you find that the POCTs were fit for purpose? What was the take home finding for the analytical validity and clinical validity?	In the discussion we have:<br>• stated the principal findings<br>• added take home finding for the analytical validity and clinical validity<br>• answer the question whether were POCTs fit for purpose
